# Phylogenomic Analysis Reveals Dynamic Evolutionary History of the Drosophila Heterochromatin Protein 1 (HP1) Gene Family

**DOI:** 10.1371/journal.pgen.1002729

**Published:** 2012-06-21

**Authors:** Mia T. Levine, Connor McCoy, Danielle Vermaak, Yuh Chwen G. Lee, Mary Alice Hiatt, Frederick A. Matsen, Harmit S. Malik

**Affiliations:** 1 Division of Basic Sciences, Fred Hutchinson Cancer Research Center, Seattle, Washington, United States of America; 2 Computational Biology Program, Fred Hutchinson Cancer Research Center, Seattle, Washington, United States of America; 3 Center for Population Biology, University of California Davis, Davis, California, United States of America; 4 Howard Hughes Medical Institute, Fred Hutchinson Cancer Research Center, Seattle, Washington, United States of America; The University of North Carolina at Chapel Hill, United States of America

## Abstract

Heterochromatin is the gene-poor, satellite-rich eukaryotic genome compartment that supports many essential cellular processes. The functional diversity of proteins that bind and often epigenetically define heterochromatic DNA sequence reflects the diverse functions supported by this enigmatic genome compartment. Moreover, heterogeneous signatures of selection at chromosomal proteins often mirror the heterogeneity of evolutionary forces that act on heterochromatic DNA. To identify new such surrogates for dissecting heterochromatin function and evolution, we conducted a comprehensive phylogenomic analysis of the Heterochromatin Protein 1 gene family across 40 million years of Drosophila evolution. Our study expands this gene family from 5 genes to at least 26 genes, including several uncharacterized genes in *Drosophila melanogaster*. The 21 newly defined HP1s introduce unprecedented structural diversity, lineage-restriction, and germline-biased expression patterns into the HP1 family. We find little evidence of positive selection at these HP1 genes in both population genetic and molecular evolution analyses. Instead, we find that dynamic evolution occurs via prolific gene gains and losses. Despite this dynamic gene turnover, the number of HP1 genes is relatively constant across species. We propose that karyotype evolution drives at least some HP1 gene turnover. For example, the loss of the male germline-restricted HP1E in the obscura group coincides with one episode of dramatic karyotypic evolution, including the gain of a neo-*Y* in this lineage. This expanded compendium of ovary- and testis-restricted HP1 genes revealed by our study, together with correlated gain/loss dynamics and chromosome fission/fusion events, will guide functional analyses of novel roles supported by germline chromatin.

## Author Summary

Our genome is comprised of two compartments. The euchromatin harbors abundant genes and regulatory information, while heterochromatin harbors few genes and abundant repetitive DNA. These characteristic features of heterochromatin challenge traditional methods of sequence assembly and molecular dissection. The analysis, instead, of proteins that localize to and often functionally define heterochromatic sequence has illuminated numerous heterochromatin-dependent, essential cellular processes, including chromosome segregation, telomere stability, and gene regulation. With the aim of increasing our sample of heterochromatin-localizing proteins, we performed a comprehensive search for new members of Heterochromatin Protein 1 gene family over 40 million years of Drosophila evolution. Our report expands this family from a modest five genes to 26 genes. Unlike the founding family members, the HP1s we describe are structurally diverse, largely restricted to male reproductive tissue, and highly dynamic over evolutionary time. Despite recurrent HP1 gene birth and death, gene numbers per species are relatively constant. These gene “replacements” likely support a dynamic biological process. We propose, and present evidence for, the hypothesis that recurrent chromosomal rearrangements drive at least some HP1 gene family dynamics observed. We anticipate that these HP1 genes will help define new heterochromatin-dependent processes in the male germline.

## Introduction

Comparative genomics has revolutionized analysis of eukaryotic genome structure, function, and evolution. Genome sequencing efforts that encompass both closely and distantly related species have led to the identification of protein- and RNA-coding genes as well as noncoding regulatory sequence on an unprecedented scale [1], [2]. This rapid progress, however, has been restricted largely to the gene-rich euchromatic genome compartment. Heterochromatin—the gene-poor, repeat-rich region found mostly near eukaryotic telomeres and centromeres—has been largely excluded from these efforts despite constituting 20–30% of human and fly genomes [3] and up to 85% of others [4]. This omission is primarily due to the highly repetitive nature of heterochromatic DNA sequence, which renders it recalcitrant to sequence assembly on which structural, functional, and evolutionary insights depend.

Heterochromatin research instead relies heavily on the analysis of the non-histone chromosomal “surrogate” proteins (reviewed in [5]) that localize to this genome compartment. This approach has illuminated roles of heterochromatin in many basic cellular and evolutionary processes such as gene regulation [6], telomere maintenance [7], [8], genome defense [9], and speciation [10], [11]. The Heterochromatin Protein 1 (HP1) gene family encodes arguably the best-known surrogate proteins for heterochromatin function. Mutant alleles of Drosophila HP1A, for example, first illuminated the essential role of heterochromatin in mitotic chromosome segregation [12]. Functional heterogeneity among HP1 paralogs also mirrors the functional heterogeneity of heterochromatic DNA. The recent identification of a female germline-specific HP1 (HP1D/Rhino) in Drosophila [13], together with its non-overlapping cytological distribution with HP1A [14], highlighted a distinct, functionally important heterochromatic compartment that encodes clusters of Piwi-bound RNAs (piRNAs) required for transposable element suppression [9]. All previously characterized HP1s localize to chromatin and, with the exception of HP1C, virtually all localize predominantly to heterochromatin [14]–[16]. We reasoned that new HP1 gene discovery via BLAST followed by a phylogenomic analysis (i.e., the prediction of gene function based on its evolutionary history in a phylogenetic tree [17]) would provide novel surrogates for exploring new heterochromatin functions. Because all annotated heterochromatin proteins are encoded in the euchromatin, our surrogate approach enables us to harness the power of euchromatic comparative genomics to illuminate diverse heterochromatin functions and evolutionary signatures.

**Figure 1:**
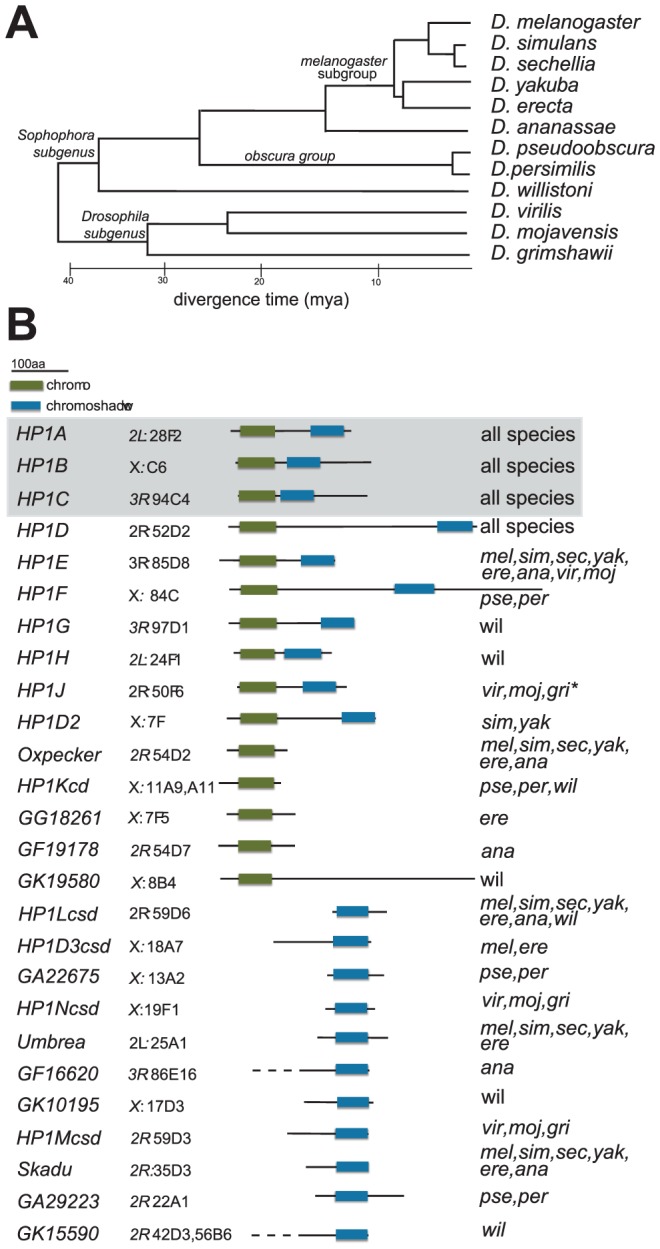
HP1 diversity in Drosophila genomes. A. Phylogeny of 12 Drosophila species, which were each queried for HP1-like genes in this study [2], [42]. Scale bar refers to the approximate divergence time between these species [2], [42]. B. Schematics of proteins encoded by the various HP1 genes in Drosophila genomes are presented alongside the HP1 gene name. Highlighted in boxes are the canonical chromo (green) and shadow (blue) domains that typify HP1 genes. Note that in some instances, we were unable to confirm the exact gene model and therefore the lengths of the N-terminal tails (these are indicated with dashed lines). We also report the *D. melanogaster* cytolocation of the gene or if the gene is absent in *D. melanogaster*, the sytenic location in the *D. melanogaster* genome based on neighboring genes. The final column reports the species in which the gene is found. Genes shaded gray represent founding HP1 gene family members that were reported in the original *D. melanogaster* genome sequencing study [42]. “*” refers to an allele that harbors a premature stop codon but conserved C-terminal sequence (Table S1, Figure S4) and predicted CD and CSD domains, consistent with a polymorphic full length gene or an incorrect base call.

**Figure 2:**
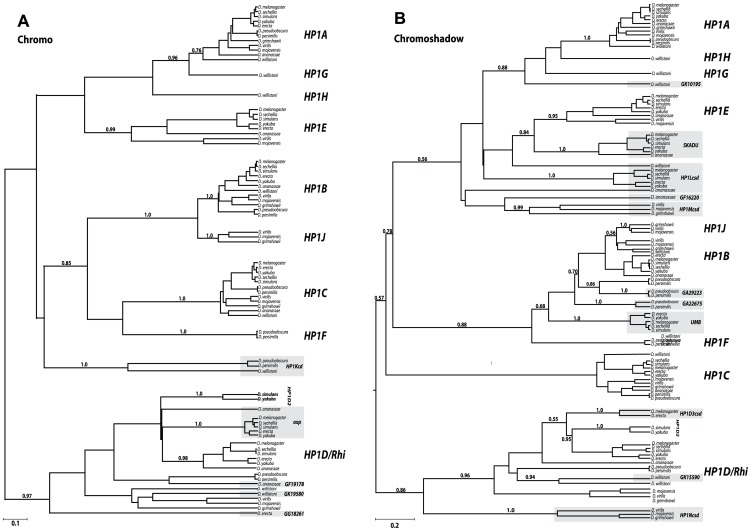
Phylogenetic relationships among the Drosophila HP1 genes. We constructed phylogenetic trees generated in BEAST (see Materials and Methods) using codon-based alignments of the Chromodomain (A) or Chromoshadow domain (B) based on a log-normal relaxed molecular clock [58]. For clarity, we only present posterior probabilities for major clade relationships rather than between orthologs of the same gene (complete trees with all posterior probability support values indicated can be found in supplemental data (Figure S5A, S5B)). In most instances orthologs grouped together with a high degree of support (exceptions, including the *HP1Lcsd* genes are discussed in the main text). Genes that are shaded gray refer to partial HP1s that encode either a chromodomain (in A) or shadow domains (in B) only. Scale bar refers to the expected number of substitutions per site.

We therefore conducted a comprehensive BLAST and phylogenomic analysis of the Heterochromatin Protein 1 gene family. Using the 12 sequenced Drosophila genomes spanning 40 million years of gene family evolution (Figure 1A, [2]), we find unexpectedly high HP1 gene numbers and structural diversity. Our analysis increases this gene family from 5 to 26 genes, including several currently uncharacterized genes in the model genetic organism, *Drosophila melanogaster*. Many of these HP1s occur in “partial” form, having lost canonical HP1 domains; nevertheless, their open reading frames have been preserved for millions of years. Unlike the three original members of the HP1 gene family, all of the newly annotated HP1s are highly species-specific and almost exclusively germline-restricted. Similar to the original members, however, we find little evidence of positive selection driving the evolution of HP1 genes using both population genetic and molecular evolution analyses. In some instances HP1 gene presence/absence correlates with karyotype evolution across this 40 million year snapshot, suggesting that large-scale chromosomal evolution may contribute to at least some HP1 birth/death dynamics. This phylogenomic analysis sets the stage for a more comprehensive dissection of germline heterochromatin function in *D. melanogaster* and other emerging model Drosophila species.

## Results

### 21 novel HP1-like genes in the Drosophila genus

Representatives of the HP1 gene family have been documented in many lineages of plants, animals, fungi and even protists, all of which harbor between one and three HP1 genes [15]. The founding family member, Heterochromatin protein 1A (HP1A) from *D. melanogaster*, was first described as a major non-histone chromosomal protein co-localizing with pericentric and telomeric heterochromatin [18], [19]. HP1A harbors an N-terminal chromodomain (CD) [20] and a C-terminal chromoshadow domain (CSD) [21] separated by a hinge (H) domain. Despite homology, the CD and CSDs are functionally divergent. The CD mediates protein-chromatin interactions via histone modifications [22]) whereas the CSD mediates protein-protein interactions, specifically recognizing a degenerate pentameric PxVxL domain in interacting proteins [23]. In some cases, the H domain binds RNA and DNA [24], [25]. We refer to the regions outside the CD and CSD as the N- and C- terminal “tails,” which are less well characterized. Since many Drosophila proteins encode chromodomains, we define HP1 gene family membership by the presence of both the CD and the CSD (“full HP1” hereafter), a CSD only (a domain exclusive to HP1 genes), or alternatively, a single CD ancestrally related to a full HP1 (see Materials and Methods). Single-domain HP1s are referred to as “partial HP1s” hereafter.

#### Full-length HP1s

Subsequent to the sequencing of the *D. melanogaster* genome, two additional HP1 genes—*HP1B* and *HP1C*— were identified (Figure 1B). These three genes alone highlight the diversity of currently known HP1 functions; whereas HP1A almost exclusively localizes to heterochromatin, HP1C localizes to euchromatin while HP1B localizes to both compartments [16]. Using tBLASTN analyses, we identified orthologs for ***HP1A*** **,** ***HP1B*** and ***HP1C*** in syntenic locations throughout the 12 sequenced species (Figure 1A), suggesting that these three HP1 genes have been preserved for >40 million years. We find that the more recently described female germline expressed ***HP1D/Rhino*** [9], [13], [14] is also preserved in syntenic locations. In contrast, the functionally uncharacterized, male germline expressed ***HP1E*** [14] is present in syntenic locations in most Drosophila species, but has been lost at least thrice–in the *D. pseudoobscura/D.persimilis, D. willistoni, and D. grimshawi* lineages (Figure 1B). *HP1E* thus represents an instance of an evolutionarily labile gene. While previously unknown for this gene family, we now find that lineage-restriction is in fact the norm rather than the exception (Figure 1B, see below).

Our tBLASTn search in the 12 Drosophila species revealed 5 additional full-length HP1-like genes that are absent from the *D. melanogaster* genome (Figure 1B). First, we identified ***HP1F***, a novel HP1 gene that is only found in *D. pseudoobscura* and *D. persimilis*. The *D. willistoni* genome harbors two previously undescribed full HP1 genes, ***HP1G*** and ***HP1H***, which are absent from all other sequenced Drosophila species (Figure 1B). Given that these genomes also lack the *HP1E* gene, we wished to rule out the trivial possibility that these ‘new’ HP1 genes simply represented a transposition of the HP1E gene into new genomic locations. We found evidence of an *HP1E* pseudogene in the syntenic location of *D. pseudoobscura* (Figure S1) and a restricted tBLASTn search (bl2seq) returned no significant hits (e-values>1.0) in *D. willistoni* or *D. grimshawii CG8861* introns, the syntenic location of HP1E in all species (data not shown). Moreover, phylogenetic analyses (presented below) demonstrate that *HP1F*, *HP1G* and *HP1H* form clades independent of *HP1E*, supporting our hypothesis that these three HP1s represent bona fide new members of the Drosophila HP1 gene family. Our analyses also uncovered ***HPIJ***, an ancient, uncharacterized HP1 paralog is retained in *D. virilis*, *D. mojavensis* and *D. grimshawi* (Figure 1B). Finally, ***HP1D2*** is retained in *D. simulans* and *D. yakuba* (Figure 1B) but lost or degenerated in *D. melanogaster* and *D. erecta* (Figure 1B, Figure S2).

#### Partial HP1 genes

The tBLASTn analyses also revealed a number of HP1 related genes that retain only the CD or the CSD domains, putatively having lost the other domain. There are five instances of CD-only HP1s and 11 instances of CSD-only partial genes, many of which occur in *D. melanogaster* (Figure 1B). Most of these partial CD- and CSD- only HP1 genes occur in distinct locations of the genome (Figure 1B). Notable exceptions are two CD-only HP1s that are found directly upstream of the *HP1D/Rhino* locus from which they are likely derived (*Oxpecker, GF19178*). Finally, at least seven partial HP1s appear to have been retained for millions of years based on their syntenic locations and intact open reading frames in multiple genomes (Figure 1B), suggesting that many of these “reduced” genes represent bona fide HP1-like genes rather than pseudogenes that have not completely degenerated. For other highly lineage-restricted HP1s (e.g, *GF19178* or *GA29223*), however, further sequencing of related species will be necessary to rule out the possibility that these coding sequences represent persistent pseudogenes. Given the short half-life of pseudogenes in Drosophila species [26], however, it appears that Drosophila genomes harbor many functional partial HP1 genes.

### Phylogenetic analyses support evidence of many ancient, undescribed HP1 lineages in Drosophila

We constructed separate CD and CSD Bayesian phylogenetic trees to evaluate support for the ancestral relationship among currently defined full-length HP1s with the 16 partial HP1 genes. This analysis enabled us to delineate previously unknown HP1 lineages and to identify the putative gene duplication events that led to some of the current diversity of HP1s in Drosophila (Figure 2A, 2B respectively). We built separate, domain-based trees for two reasons. First, prior studies had suggested the possibility that the phylogenetic histories of previously known CD and CSD are not always congruent [27]. Fusions of a CD and CSD from different HP1 lineages or evolutionary rate heterogeneity between the two domains may account for this observation. Second, we wished to analyze the origin of multiple CD-only and CSD-only partial HP1 genes, which would not have been possible on a combined ‘CD and CSD’ phylogeny.

#### Full-length HP1s

We find that four of the five new full HP1s represent well-supported sister clades of previously known family members. The *D. willistoni*-restricted ***HP1G*** shares a common ancestor with an ancestral *HP1A* based on both the CD and the CSD trees, while the *D. pseudoobscura/D. persimilis* lineage-restricted ***HP1F*** CD groups with the HP1B/HP1C clade and its CSD groups with HP1B clade exclusively. We also find support for an ***HP1H***, *HP1G*, and *HP1A* clade on the CSD tree; however, we find only weak support for this relationship on the CD tree, possibly due to rapid evolution of the HP1H chromodomain. Based on the CD and CSD phylogenies, it appears that the duplication of ***HP1J*** from an HP1B-like ancestor either predated the Drosophila genus followed by subsequent loss in the Sophophora subgenus (CD phylogeny- Figure 2A) or originated exclusively in the Drosophila subgenus (CSD phylogeny- Figure 2B). The full-length *HP1F, HP1G, HP1H, and HP1J* likely represent new HP1 lineages. In contrast, ***HP1D2*** clusters consistently within the *HP1D* clade, supporting a relatively recent duplication event leading to this paralog. Further phylogenetic sampling is required to fully resolve the duplication events leading to *HP1G*, *HP1H*, *HP1F*, and *HP1J*, which may pre- or post- date the 40 million year old ancestor. We observed no daughter or sister clades of *HP1C*, the only known HP1 that localizes almost exclusively to euchromatin (Smothers and Henikoff 2001).

The partial HP1 genes fall into three classes—those that cluster within full HP1 clades, those that share a more distant common ancestor with the full HP1 clade (a sister lineage to a full HP1 clade), and finally, those that represent a completely new HP1 lineage that likely emerged prior to the origin of the Drosophila genus, more than 40 mya. As an example of the first class, the *D. pseudoobscura/persimilis* gene *GA29223* falls within the *HP1B* clade, and is actually most closely related to this lineage's *HP1B*, implicating a recent duplication event (Figure 2B). Similarly, *GK15590* appears to have recently duplicated from *HP1D* (Figure 2B). Representative of the second class, *Oxpecker* (Figure 2A) shares a more distant common ancestor with the *HP1D* clade as does *Skadu* and *HP1E*. These sister clades to full HP1s likely emerged between 10 and 20 mya. Finally, deep branching clades such as *HP1Kcd*, *HP1Lcsd*, *HP1Mcsd*, and *HP1Ncsd* represent the third class; their phylogenetic position suggests that these lineages may share an even more distant ancestor with all known HP1s, apparently absent from our phylogenetic trees. Alternatively, this third class of partial HP1 genes might be evolving very rapidly, obscuring their true phylogenetic position. This may especially be the case for *HP1Lcsd*, which we propose harbors orthologs in *D. ananassae* and *D. willistoni* by synteny, but this orthology is not evident in the phylogenetic analysis (Figure 2B).

Curiously, all CD-only partial HP1s derived from a full HP1 are related to *HP1D/Rhino*. In contrast, the abundant CSD-only HP1s share a most recent common ancestor with a diversity of HP1- like genes – *HP1B*, *HP1D*, *HP1G*, and *HP1E*, and possibly others that cannot as yet be assigned with high confidence. One explanation for this non-random pattern, assuming that the duplication rate across HP1s is constant, is that the CD that typically encodes the property of binding specific chromatin modifications may interfere with the evolution of novel HP1 genes or functions. The *HP1D/Rhino*-derived CD-only HP1s, the exceptions to this pattern, may be retained at least in part due to a genome defense-related function similar to their putative parent (9, 14). Alternatively, the 3′ bias in retrogene formation may explain this apparent preferential retention of the CSD. More generally, we observed no instances of a partial HP1 that shares a most recent common ancestor with a paralogous, partial HP1. This suggests that unlike full-length HP1s, partial HP1s do not seed additional paralogs, at least in Drosophila. Finally, we found neither sister nor daughter clades of *HP1C* among full or partial HP1 genes. Thus, the only exclusively euchromatin-localizing Drosophila HP1, HP1C, has not given rise to paralogous lineages, reinforcing our assumption that heterochromatin function drives this gene innovation in Drosophila HP1 genes. In total, our analyses reveal an unprecedented number and diversity of HP1s in this once narrowly defined gene family.

### HP1 innovation in the Drosophila male germline

**Figure 3:**
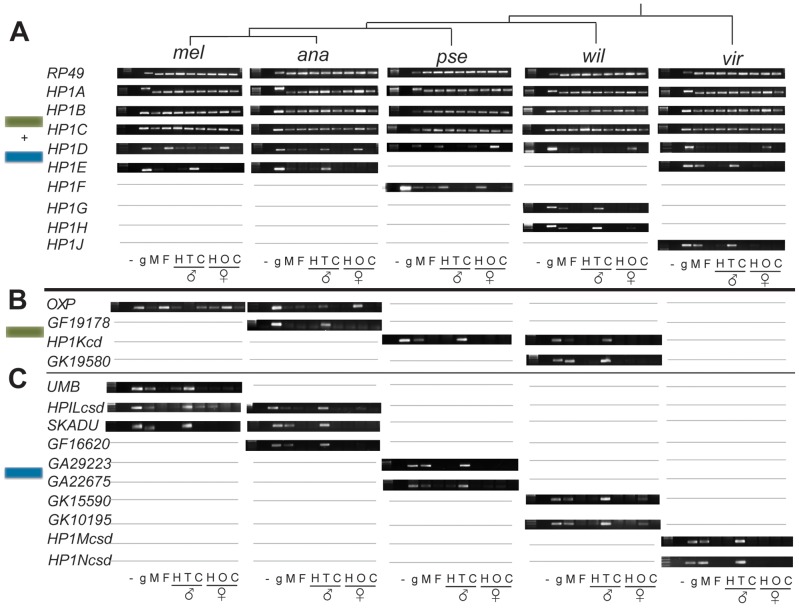
Expression patterns of Drosophila HP1 genes. RT-PCR analysis on several adult tissues from male and female Drosophila from each of 5 species. RP49 represents a control locus. “UMB:” *umbrea*, “OXP”: *oxpecker*, “-”: no DNA/RNA control; “g”: genomic DNA, “M”: whole male, “F”: whole female; “H”:head; “T”: testis, “C”: carcass (gonadectomized, headless individuals); “O” ovaries. Gray lines refer to the absence of the gene in the particular species. We present the analyses for full-length HP1 genes in (A), for partial CD-only HP1s in (B) and for partial CSD-only HP1s in (C).

Since most of the HP1 genes we have identified are completely uncharacterized, we investigated their transcript levels across adult tissues. We prepared cDNA from six tissue types in five species of Drosophila- *D. melanogaster*, *D. ananassae*, *D. pseudoobscura*, *D. willistoni* and *D. virilis* (Figure 3). The selected subsample of species maximized the number of newly defined HP1s analyzed (Figure 1B). Consistent with previous results, we found that *HP1A*, *HP1B*, and *HP1C* are expressed ubiquitously across sampled adult tissues. This expression profile is conserved across all 5 species assayed (Figure 3A). In addition, *HP1D/Rhino* is expressed predominantly in the ovaries of all species, and is also weakly expressed in *D. ananassae* testes.

**Figure 4:**
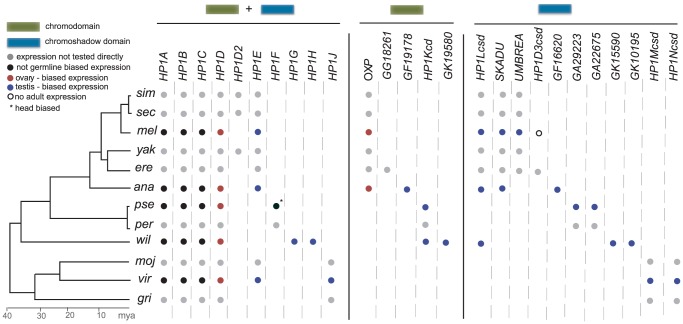
HP1 gene compendia in the 12 Drosophila species. We present the summary of all HP1-like genes that were identified in our evolutionary screen. These include the five previously known *HP1A* through *HP1E* but also include 21 additional HP1 genes identified in this study. Unlike *HP1A* through *HP1D*, which are present throughout Drosophila phylogeny, many HP1 genes are present or lost in a lineage- or even species-specific fashion. Summarizing the expression patterns in five Drosophila species (Figure 3 above), we report either ubiquitous expression (i.e., not germline biased) or ovary- or testis-biased expression. Most of the genes we have identified have a germline- and specifically testis-biased expression. *HP1F* (*) appears to be exclusively expressed in *D. pseudoobscura* heads. Open circles refer to genes where we did not find evidence for adult-specific expression.

In striking contrast, virtually all lineage-restricted HP1 genes reported in our phylogenomic analysis exhibit germline and primarily testes-restricted expression (Figure 3A, 3B, summarized in Figure 4). The only exception is *D. pseudoobscura*'s *HP1F*, which is expressed in male and female heads only. *HP1E*, *HP1G*, *HP1H*, *HP1J*, *HP1Kcd*, *GF19178*, *GK19580*, *Umbrea*, *HP1Lcsd*, *Skadu*, *GF16620*, *GA29223*, *GA22675*, *GK15590*, *GK10195*, *HP1Mcsd* and *HP1Ncsd* are all predominantly expressed in testes. Although we have not formally ruled out exclusive expression in the somatic cells of the testis sheath, it is likely that this enrichment reflects specific expression in the germline (M. Levine, unpublished data). *Oxpecker* is the only partial HP1 expressed in the ovary while *HP1H* is expressed in both testis and ovary, but only weakly in the latter. We did not recover robust evidence for expression of *HP1D3csd* in *D. melanogaster* adults (data not shown). Together, our results argue that constant innovation in the HP1 gene family has been driven by lineage-specific requirements in the Drosophila male germline.

### Molecular population genetics and evolution of germline-expressed HP1 genes

We next investigated the possibility that positive selection is associated with this recurrent innovation at the level of whole HP1 genes. A significant excess of positive selection signatures at testis-biased genes is routinely observed (reviewed in [28], [29]), consistent with pervasive sexual selection, host-pathogen interactions, and/or segregation distortion acting on those loci encoding products active in male reproductive tissue. Moreover, very young genes of comparable age to the young HP1s harbor an excess of such positive selection signatures [30], [31]. Finally, previously published evidence of positive selection acting on the ovary-restricted *HP1D/Rhino* [14] implicated an unusual, specialized function for this HP1 gene which was borne out by later functional analyses [9]. To test the hypothesis that pervasive positive selection, and possibly genetic conflict, drives DNA sequence evolution of the germline-restricted, evolutionarily labile HP1s, we performed a comprehensive molecular population genetic and evolution analysis of DNA sequence polymorphism and divergence using publically available datasets of 44 *D. melanogaster* genomes and the full genome sequences of up to 9 close relatives (see Materials and Methods). Several of these parameter estimates also enabled us to test whether these newly described HP1s are functional. We included the HP1 gene family members that have been previously functionally characterized (*HP1A, HP1B, HP1C, HP1D*). In particular, the previously published strong signature of positive selection found at *HP1D/Rhino* [14] makes this locus a convenient “positive control.”

We focused on those HP1s that occur in *D. melanogaster* for which we have the most population genomic data, many closely related sequenced genomes, and the highest tractability for future functional analyses. We first investigated codon usage bias. The presence of only a narrow subset of redundant codons in coding sequence is consistent with gene function [32]. For each HP1 found in *D. melanogaster*, we estimated the “effective number of codons” or “ENC”, where 1 is the most biased and 61 is the least. In general, we observe homogeneity of low ENC estimates in *HP1A, HP1B, and HP1C*, while there is striking heterogeneity among the remaining HP1-like genes (Table 1). Moreover, elevated ENC estimates (low codon usage bias) for *Skadu* and *HP1Lcsd* places them in the 99^th^ percentile of all *D. melanogaster* genes [33], perhaps indicating loss of functional constraint. We observed a similar trend of heterogeneity in the new HP1 members for the ratio of nonsynonymous to synonymous π, an estimate of intraspecific sequence diversity. An excess of nonsynonymous mutations (and therefore a high ratio assuming typical synonymous π—a signature of pseudogenes) may be consistent with a loss of functional constraint. *HP1Lcsd* is in the 99^th^ percentile for both the π ratio and ENC, which might indicate a loss of constraint at least along the *D. melanogaster* lineage despite its retention across more than 30 million years of Drosophila evolution.

**Table 1:** Results of the population genetic analyses of HP1 genes that occur in *D. melanogaster*.

	Codon Usage Bias		Syn+Nonsyn Sequence Diversity						McDonald-Kreitman Test						
gene	ENC mel/sim/yak	%ile	ave n	#codons	NSπ	Sπ	π ratio	%ile	#codons	NSfix	Sfix	NSpoly	Spoly	NI	FETpval
*HP1A*	47/48/45	43.1	38.5	206	0.000	0.004	0.08	50.7	206	6	13	6	9	1.44	0.72
*HP1B*	40/40/36	9.8	37.6	240	0.001	0.013	0.11	60.5	239	1	11	2	17	1.29	1.00
*HP1C*	39/39/40	8.0	36.5	237	0.000	0.011	0.02	22.0	237	7	9	6	25	0.31	0.10
*HP1D/Rhino*	59/59/56	93.6	40.8	418	0.004	0.009	0.41	93.2	413	101	44	34	20	0.74	0.40
*HP1E*	46/61/61	31.1	35.5	174	0.004	0.004	0.95	98.2	172	14	23	15	12	2.05	0.21
*Skadu*	61/58/59	99.9	37.3	133	0.007	0.018	0.39	92.4	133	13	8	11	10	0.68	0.76
*Umbrea*	56/49/58	86.5	38.5	106	0.003	0.003	0.84	97.9	102	28	8	4	3	0.38	0.35
*HP1Lcsd*	61/49/56	99.9	34.6	83	0.001	0.001	1.13	98.7	81	23	11	6	3	0.96	1.00
*Oxpecker*	59/45/53	96.1	40.4	84	0.001	0.005	0.14	70.3	84	7	15	6	5	2.57	0.27
*HP1D3csd*	55/-/52**	81.7	37.0	173	0.003	0.007	0.38	92.0	88	36	12	16	7	0.76	0.78

ENC = effective number of codons, Syn = synonymous, Nonsyn = nonsynonymous, %ile = percentile, ave n = average # alleles, NS = nonsynonymous, S = synonymous, π ratio = NSπ/Sπ, poly = #polymorphisms, fix = # fixations, NI = neutrality index, FETpval = Fisher's Exact Test probability value.

To test for heterogeneity in rates of DNA sequence evolution between species (*D. melanogaster* and *D. simulans*) among the founding HP1 family members, we calculated pairwise dN/dS ratios using the PAML suite of programs. These estimates are also consistent with substantially different rates of evolution between the founding members and most newly described HP1s (Table 2). We found that *HP1A*, *HP1B*, and *HP1C* have evolved between *D. melanogaster* and *D. simulans* at substantially slower rates than most germline-restricted HP1s. At the other extreme, the dN/dS for the coding sequence of *HP1D/Rhino* and *Skadu* are in the 99^th^ percentile of all *D. melanogaster* genes, while *HP1Lcsd* and *Umbrea* are in the 95% [33]. The codon bias and π ratio estimates for *HP1Lcsd* may be consistent with elevated dN/dS driven by a loss of constraint along the *D. melanogaster* lineage but *Skadu* and *Umbrea* may be evolving under positive selection (see below), as previously shown for *HP1D/Rhino* [14].

**Table 2:** Results from the molecular evolution analysis of genes that occur in *D. melanogaster*.

gene	dN/dS	dN	dS	%ile
*HP1A*	**0.16**	0.010	0.06	75.4
*HP1B*	**0.07**	0.002	0.03	49.5
*HP1C*	**0.06**	0.006	0.10	45.0
*HP1D/Rhino*	**1.29**	0.084	0.065	99.6
*HP1E*	**0.11**	0.022	0.20	64.5
*Skadu*	**1.81**	0.025	0.01	99.7
*Umbrea*	**0.71**	0.079	0.11	98.0
*HP1Lcsd*	**0.53**	0.080	0.15	96.2
*Oxpecker*	**0.08**	0.013	0.17	54.0
*HP1D3csd*	**n/a**	n/a	n/a	n/a

The dN/dS refers to a *D. melanogaster-D. simulans* pairwise calculation.

To test for a history of recurrent adaptive protein evolution at these and the remaining loci, we performed a McDonald-Kreitman test (20) using polymorphism data for both *D. melanogaster* and *D. simulans* and the divergence estimates between them. Homogeneity of fixations (differences between species) and polymorphisms (differences within species) for synonymous and nonsynonymous sites is consistent with neutral expectations, while an excess of nonsynonymous fixations between species is consistent with a history of recurrent positive selection. We found that not a single HP1 analyzed harbors the signature of recurrent positive selection (Table 1). One qualifier of this analysis is that a locus must experience positive selection at many sites to generate enough power to reject neutrality. This is especially relevant to *HP1D/Rhino*, for which a history of positive selection has been described, but only on the chromoshadow and C-terminal tail between these species, which would not emerge from this whole-gene analysis and with so little publically available *D. simulans* polymorphism data. Moreover, several genes harbor exceptionally few synonymous polymorphisms, further weakening our statistical power.

Given these limitations, we subjected the same set of genes to a PAML analysis, which has additional power to detect recurrent positive selection acting at sequence encoding only a single domain. As expected, we find a significant signature of positive selection at *HP1D/Rhino* (Table 3). The CSD-only HP1, *Umbrea*, harbors equally strong evidence of recurrent adaptive evolution. However, we found no evidence of positive selection (Table 3) for any other germline-restricted HP1—both those conserved across the 40 million years of evolution (e.g., *HP1A* and *HP1B*) and those that are relatively young (e.g., *Oxpecker*, *HP1Lcsd*, *Skadu*). This finding was particularly surprising for *HP1E*, the only full-length HP1 expressed predominantly in male reproductive tissues that we previously hypothesized to serve a functionally analogous role to the ovary-restricted, piRNA defense pathway member, *HP1D/Rhino* [27]. Our findings are consistent with *HP1E* and *HP1D/Rhino* evolving under different evolutionary forces. In summary, molecular population genetic and evolution analyses are consistent with mostly purifying selection and loss of constraint acting on the newly described HP1s that occur in *D. melanogaster*.

**Table 3:** PAML analysis results of genes that occur in *D. melanogaster*.

gene	species	#codons	log ratio	p-val
*HP1A*	*mel,sim,sec,ere,yak,tak,bia,ele*	206	0.00	1.00
*HP1B*	*mel,sim,sec,ere,yak,tak,bia,ele,fic*	660	5.56	0.06
*HP1C*	*mel,sim,sec,ere,yak,tak,bia,ele,fic*	356	0.19	0.91
*HP1D/Rhino*	*mel,sim,sec,ere,yak,tak,bia,ele*	257	12.36	**0.00**
*HP1E*	*mel,sim,sec,ere,yak,tak,bia,ele,fic*	144	0.17	0.92
*Skadu*	*mel,sim,sec,ere,yak,tak,bia,ele,fic*	125	1.14	0.56
*Umbrea*	*mel,sim,sec,ere,yak,tak,ele,fic*	216	13.44	**0.00**
*HP1Lcsd*	*mel,sim,sec,ere,yak,tak,bia*	66	2.70	0.26
*Oxpecker*	*mel,sim,sec,ere,yak,tak,bia,ele*	60	0.00	1.00
*HP1D3csd*	*mel, ere*	173	n/a	n/a

*mel* = *D. melanogaster*, *sim* = *D. simulans*, *sec* = *D. sechellia*, *yak* = *D. yakuba*, *ere* = *D. erecta*, *tak* = *D. takahashii*, *bia* = *D. biarmipes*, *ele* = *D. elegans*, *fic* = *D. ficusphilia*.

### The “revolving door” of HP1 gene family evolution

The relatively constant HP1 gene number in any given species combined with pervasive birth-death dynamics across the broader tree is consistent with a “revolving door” model [34], where one gene emerges along a lineage as another is lost. The pattern is readily apparent in Figure 4. Non-orthologous CSD-only genes, for example, occur in each species or clade harboring at least one exclusive gene of this class (*Umbrea, Skadu, HP1Lcsd, GA29223, HP1Ncsd*). *HP1E* is found in eight of the 12 species. In the four species where *HP1E* is absent, at least one additional lineage-restricted, full HP1 is present. The *D. pseudoobscura/D. persimilis* lineage has *HP1F*, *D. willistoni* has *HP1G* and *HP1H*, and *D. grimshawii* has *HP1J*. Even across classes, we observe this pattern – the *HP1D/Rhino*-derived genes *HP1D3csd* and *HP1D2* are retained in a mutually exclusive manner (Figure 1B). These lineage-restricted HP1s may support a common but dynamic biological function that, like these genes, may be turning over repeatedly across the 40 million years examined.

The 40 million year snapshot captured by the 12 Drosophila genomes harbors diversity at all levels of biological organization [2]. Particularly relevant to proteins that localize to chromatin is the diversity of heterochromatin content and chromosomal distribution. Moreover, chromosomal fissions and fusions, as well as satellite expansions and contractions, result in changes to chromosomal environment, e.g., spreading or retreating of heterochromatin-euchromatin boundaries [35]. Heterogeneity in these features abounds across Drosophila evolution [36], [37]. Chromosomal rearrangements can therefore serve as proxies for changes in heterochromatin content and distribution. Similar karyotypes, however, can also belie changes in heterochromatin content; satellite DNA content comprises 44% and 2% of *D. virilis* and *D. mojavensis* genomes respectively despite a similar karyotype [36]. We wondered if the alternative retention of HP1 genes correlates with the known karyotype and heterochromatin distribution evolution across the 12 genomes.

**Figure 5:**
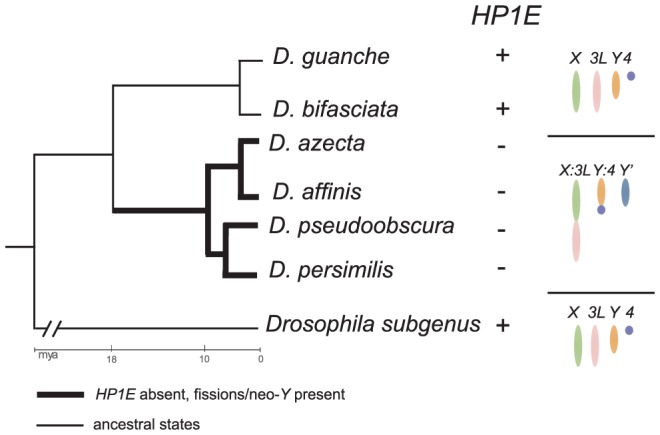
Delineating HP1E loss in the *obscura* group. We amplified the syntenic region of HP1E in the obscura group and successfully identified intact *HP1E* genes from *D. guanche* and *D. bifasciata*. We found highly pseudogenized versions of *HP1E* in *D. azteca*, *D. affinis* (Figure S3). These latter four species also share dramatic karyotypic changes specific to this lineage including an *X:3L* fusion, a *Y:4* fusion and a neo-*Y* (indicated as *Y*′ in figure, note that *3L* and *4* = elements “D” and “F”, respectively). Thus, to the level of resolution possible from the available species, *HP1E* loss coincided with the karyotypic changes in the *obscura group*. The *HP1E* cytolocation on chromosome *3R* (element “E”), post-karyotype evolution, is apparently undisrupted.

High resolution dating of karyotype evolution in the *obscura group* [38] represents an opportunity to evaluate this hypothesis. Between 11 and 18 mya, an ancestor within the *obscura* group evolved an *X*-D element fusion ([39], element “D” = *3L* in *D. melanogaster*), a neo-*Y* chromosome [40] putatively derived from the D element, and a *Y*∶F chromosome fusion ([38], the *F* refers to the *4* ^th^ chromosome in *D. melanogaster*). These fusion events combine chromosomes with qualitatively different complements of non-histone euchromatin and/or heterochromatin proteins, in addition to generating a neo-*Y* that has acquired heterochromatin characteristics typical of the ancestral Drosophila *Y* chromosome [40]. We therefore undertook the sequencing of the *HP1E* locus from the *obscura group*—*D. affinis*, *D. azteca*, *D. guanche*, *D. bifasciata*—to compare with our *D. persimilis* and *D. pseudoobscura* data. Strikingly, we find that the *HP1E* loss event dates precisely to the ancestral lineage in the obscura group that underwent the chromosomal rearrangements (Figure 5, Figure S3). *D. affinis* and *D. azteca*, which share the derived karyotype found in *D. pseudoobscura* and *D. persimilis*, harbor a highly pseudogenized *HP1E* in the syntenic location (Figure 5, Figure S3). Although only a correlation, this observation suggests the possibility that selection at *HP1E* was relaxed in association with this karyotype evolution. Alternatively, the HP1E loss may have favored the fixation of one or more of these chromosomal rearrangements (see below). Analysis of *HP1E* function, guided by this association of gene loss with a major sex chromosome evolution event, will help further illuminate the forces driving its recurrent degeneration.

Given the vast evolutionary distance between sampled species, the 12 genomes are admittedly suboptimal for a more general analysis. The well-described karyotypic diversity, however, has the power to at least highlight associations worthy of further fine scale analyses. For example, species in the Drosophila subgenus (*D. virilis, D. mojavensis, D. grimshawii*) exclusively share the ancestral “five-rod” arrangement [37]. They also share many lineage restricted HP1s (*HP1J, HP1Mcsd, HP1Ncsd*) despite spanning virtually equivalent evolutionary distance across the whole tree (∼30 my). We observe the myriad *HP1D/Rhino*-derived CD-only HP1s only in lineages with fused Muller B and C elements (*D. melanogaster, D. simulans, D. sechellia, D. yakuba, D. erecta, D. ananassae, D. willistoni*) rather than being randomly distributed across the tree. Notably, one of the best-characterized, germline-expressed piRNA clusters [41] resides near the centromere of element C and these partial HP1s are actually *independently* derived from the CD of HP1D/Rhino, a piRNA defense protein that localizes to this cluster (9). Finally, two independent HP1E loss events date to branches that have undergone independent Muller element *X*-D fusions and dot chromosome fusions to the ancient *Y* and ancestral *3R* in *D. pseudoobscura* and *D. willistoni*, respectively.

## Discussion

The genome sequence of *Drosophila melanogaster*, published in 2000, served to expand the HP1 gene family from one to three members—*HP1A* (the founder), *HP1B*, and *HP1C* [42]. A family size of three is currently the maximum number of HP1s identified in any eukaryotic lineage outside of Drosophila. For example, mammalian genomes harbor *HP1α*, *HP1β* and *HP1γ*, which are derived from vertebrate-specific duplications of an ancestral *HP1B*-like gene [15], [27]. The early Drosophila HP1 family members are transcribed ubiquitously in both sexes, have persisted for over 40 million years of Drosophila evolution, and participate in many chromatin-dependent, somatic cellular functions (reviewed in [27]). Unlike the founders, the new HP1 gene family members exhibit pervasive lineage restriction, domain degeneration, and predominant germline expression (summarized in Figure 4).

Across the 40 million year snapshot examined here, our analysis expands the Drosophila HP1 gene family from 5 to 26 members. If anything, this staggering increase in HP1 gene diversity is likely to be an underestimate. For instance, with our iterative BLAST search strategy we would not be able to detect CSDs or CDs that are highly diverged from all of the Drosophila HP1 genes identified in this study. Moreover, our methods would not detect HP1-derived genes that have only retained the original hinge or tails due to degeneration/loss of both the CD and CSD. Finally, we observed a somewhat smaller number of partial HP1 genes in species that share a more distant common ancestor with the well-annotated *D. melanogaster*, which might indicate that genome assembly gaps influence HP1 discovery biases. Nevertheless, our search represents the most exhaustive to date and proved substantially more powerful than previous genome-wide scans. Indeed, automated gene prediction algorithms and annotation software failed to predict coding sequences and/or identify many genes as HP1s, even in the well-annotated genome of *D. melanogaster*. Poor homology to known genes, some exceptionally short protein lengths especially for the partial HP1s, and extensive divergence/degeneration of the typically conserved CD and CSD domains may have concealed these HP1s from automated, DNA sequence-based, genome-wide methodology (*D. melanogaster* R4.3)

Using a phylogenomic approach, we set out to identify new surrogates for dissecting chromatin, and specifically, heterochromatin function and evolution. Given that all previously described Drosophila and non-Drosophila HP1s localize to chromatin [15], [27], we expect that the new full-length HP1s also encode non-histone chromosomal proteins. We also predict that the CD-only partial HP1s localize to chromosomes given that the CD specifically recognizes histone modifications [22], [43]. Although the localization of partial HP1s that harbor only a CSD (a protein:protein interaction domain) is harder to predict, virtually all CSD-only HP1s share a common ancestor with a CSD that interacts with chromosomal proteins [15], [27]. This phylogenetic signature is consistent with chromatin localization even for these proteins. This prediction holds for the only cytologically characterized CSD-only protein, *Umbrea*, which has been shown to localize to heterochromatin [44] and we have shown more specifically localizes to centromeres (B. Ross and H. Malik unpublished, [27]). Confirming *heterochromatin* localization for each new HP1 will require detailed cytological analysis. Nevertheless, it is intriguing that none of the newly identified HP1 genes share a most recent common ancestor with *HP1C*, the only well-characterized HP1 that localizes exclusively to euchromatin. In other words, only the heterochromatin-localizing HP1s–HP1A, HP1B, and HP1D—emerged as parental or sister clades to the new HP1s for which we observe significant phylogenetic support.

Whereas the molecular dissection of early HP1 members has illuminated the myriad heterochromatic and some euchromatic functions in somatic cells, the new surrogates we describe here will serve instead as guides in dissecting the germline. With the exception of HP1F, all newly described HP1 members are expressed predominantly in germline tissue and all are highly lineage-restricted, implicating species-specific specialization and possible functional replacements (Figure 4). However, unlike most testis- and lineage- restricted, young Drosophila genes [28], [30], [31], we found no evidence of positive selection in most genes subjected to close evolutionary analyses. The results implicate biological functions that turnover on relatively longer time scales than the intragenomic conflict that putatively drives positive selection at *HP1D/Rhino* [9], [14] and other testes specific processes [45]–[47]. The absence of a positive selection signature is particularly surprising for *HP1E*, which is the only full-length HP1 paralog in *D. melanogaster* expressed predominantly in testes. Indeed, we predicted that *HP1E* was the male functional analog of the female genome defense paralog, *HP1D/Rhino*, possibly supporting the sexually dimorphic piRNA pathway in males [27]. These data weaken the prediction that *HP1E* acts at the interface of host-TE interactions and may instead functionally replace *HP1A* in the male germline, as has been previously suggested [48]. A better candidate male analog might be any of the highly lineage-restricted partial HP1s *GK19580, GF19178, and GG18261* that are constantly birthing from the *HP1D/Rhino* CD and may encode male genome-defense proteins that constantly turnover in response to TE turnover. Given the restricted subcellular localization of *HP1D/Rhino* to piRNA clusters [9], [27], we speculate that these *HP1D*-derived genes may also be involved in germline defense.

While the role of *HP1E* in germline function remains undiscovered, its phylogenetic signature may be illuminating. We had initially predicted that *HP1E* was functionally replaced by *HP1G* and/or *HP1H* in *D. willistoni* and by *HP1F* in *D. pseudoobscura* [27]. To our surprise, however, *HP1F* is expressed in male and female heads only, weakening this hypothesis. *D. pseudoobscura* is the only species represented in the 12 sequenced genomes where a testis-restricted, full length HP1 is absent (see summary in Figure 4). It is also the only species without the ancestral Drosophila *Y*; instead it now has a neo-*Y* chromosome [40]. Moreover, the date of the *HP1E* loss (and potentially *HP1F* gain) precisely matches this karyotypic change. We speculate that the failure of *D. pseudoobscura* to “replace” *HP1E* with a full-length, testis-expressed HP1 may be related to the evolutionary dynamics of *Y* chromosomes in Drosophila species. We predict that *HP1E* interacts with (ancestral) *Y*-linked heterochromatin in species like *D. melanogaster*. Loss of this heterochromatin may have obviated the necessity for HP1E retention in *D. pseudoobscura*.

These data put forth a general hypothesis that a species' compendium of chromosome-localizing proteins may evolve following major chromosomal rearrangements and/or heterochromatin-euchromatin boundary shifts. This evolutionary prediction is consistent with the observation that EMS-induced chromosome fusions result in phenotypes modulated by non-histone heterochromatin proteins. For example, two independently-derived *X*:*4* fusion mutants exhibited sex chromosome nondisjunction and aberrantly low transcriptional output from the *X*-linked, heterochromatin-embedded rDNA locus [49]. Although the rDNA locus was intact in both cases, these mutants nonetheless manifested the classic rDNA deletion phenotype (*bobbed*) that also variegates with heterochromatin dosage. This kind of heterochromatin-dependent gene regulation is enhanced and suppressed by many classes of heterochromatin surrogate proteins. The gain and loss of heterochromatin-localizing proteins over evolutionary time may therefore prove to be recurrent events following naturally occurring chromosome fissions and fusions as well as other events driving expansions and contractions of heterochromatin.

Alternatively, the birth and death of HP1 gene family members may drive karyotype evolution. Selfish genomic elements that cheat meiosis are often associated with chromosomal rearrangements that physically link segregation distorter loci and their enhancers (reducing recombination frequency between them). An SD-*enhancing* HP1 that is linked to the fused chromosome might favor the retention of a rearrangement involving a drive locus. In contrast, an unlinked HP1 *suppressor* of drive, once fixed, would precipitate drive system breakdown and ultimately, HP1 gene degeneration—a model consistent with the HP1 revolving door we observe.

Our phylogenomic analysis of the HP1 gene family over 40 million years of Drosophila evolution introduces many genes with the exciting potential of illuminating germline chromatin-dependent biology. Newly developed tools described for the non-melanogaster Drosophila species [50], [51] will also aid the functional dissection of HP1 genes not found in *D. melanogaster*.

## Materials and Methods

### Bioinformatic analyses

We used the chromodomains (CD) and chromoshadow domains (CSD) of the five previously described HP1 gene family members (HP1A, HP1B, HP1C, HP1D, HP1E, www.flybase.org) as queries in tBLASTn searches [52] of the 12 sequenced Drosophila genomes ([2], Figure 1A). All newly identified CD- and CSD- bearing genes (identified initially by e-value less 0.1) were then culled by Prosite prediction of each domain (www.expasy.org/prosite/) or ruled out due to homology to a known non-HP1 gene in *D. melanogaster*. CSDs are exclusive to the HP1 family and indeed no CSD query from a newly identified HP1 returned a BLAST hit with an E-value less than 1.0 to a non-HP1 gene. CD occur in many non-HP1 proteins, such as Polycomb, Su(var)3–9, and MSL3 [53]. We report the consistently higher e-values for hits to non-HP1 proteins than to the best BLAST hits, which were exclusively previously identified HP1s (Table S3). These hits subsequently served as queries for new searches of the 12 genomes. This strategy was iterated with both HP1 CDs and CSDs until no new CSDs were recovered or hits to only non-HP1 CDs were recovered. We classified CD-only hits as an HP1 family member only for those genes that share a most recent common ancestor with a full length (chromo- and chromoshadow- containing) HP1 clade with high significance (posterior probability >0.95, see below). The only exception was the newly described *HP1Kcd*, which is a CD-only lineage of HP1 that represents the remnants of an ancestral HP1 no longer present in this 40 million year snapshot or alternatively, a lineage whose rapid evolution obscures its phylogenetic relationships within Drosophila (see Results). In this exceptional instance, BLAST hits to paralogous HP1s only in Drosophila and to *Anopheles gambiae HP1A* outside of Drosophila support our classification of *HP1Kcd* as an HP1 family member. In contrast to CDs, we classified all hits harboring a CSD as an HP1 gene family member since CSDs are an exclusive feature of HP1s [21]. Given that *D. melanogaster* served as the scaffold for genome assemblies, we anticipate that we likely missed proportionately more paralogs from genomes that share an increasingly distant common ancestor with this model species. However, our ability to identify new HP1 genes unique to individual species other than the well-annotated *D. melanogaster* suggests that this compendium is exhaustive. We cannot rule out, however, that unassembled stretches within the 12 genomes harbor HP1 gene family members that are not reported below. Moreover, any HP1s genes that retain only the “hinge” region (between the CD and CSD) or the “tails” (outside the CD and CSD) would be missed by our search strategy.

### Nomenclature

Since several genes that we have identified and validated represent either unannotated genes or annotated genes that have yet to be named (see Table S1 for complete list of flybase IDs or coding sequences if unannotated), we adopted a nomenclature scheme where orthologs are identified with the same gene name only if orthology is supported by both phylogenetic analyses and syntenic location (thus, *HP1A* in *D. melanogaster* and all other Drosophila species). One exceptional gene is *HP1Lcsd*, which occurs in the syntenic location in the *D. melanogaster* subgroup (Figure 1A, 1B), *D. ananassae*, and *D. willistoni*, but fails to cluster phylogenetically for the latter two species. We tentatively refer to all of these genes as *HP1Lcsd* given the low probability of two independent insertion events of a CSD-only HP1 into the same location. In cases where a newly defined HP1 clusters phylogenetically **within** a broadly distributed HP1 but the synteny criterion is not met, we refer to these genes as potential paralogs (*HP1D2* for “full” HP1s and *HP1D3csd* for a CSD only gene, for example). In cases where no consistent phylogenetic relationships or synteny can be established, or the common ancestor among a previously known and undescribed clade appears to pre-date the 40 million year old ancestor, we refer to these as ‘new’ clades of HP1 genes with a separate letter designation. Thus, we have designated these genes from *HP1A* to *HP1Ncsd*, skipping letter “I” for clarity. If a new partial gene is represented in only a single species (or only the *D. pseudoobscura/D.persimilis* lineage), we used the flybase.org gene name (e.g., *GA22675*). Finally, since the partial HP1s *HP6/Umbrea*, *Skadu* (‘Skadu’ is the Afrikaans word for ‘shadow’), and *Oxpecker* have been referred to previously in the literature [27], [54]–[56], we retain these names. *HP1E* sequences amplified from *D. affinis*, *D. azteca*, *D. guanche* and *D.bifasciata* have been submitted to Genbank under accession numbers JQ889685–JQ889688.

### Phylogenetic analyses

We inferred ancestral relationships among orthologs and paralogs from CD or CSD phylogenetic trees generated by the Bayesian MCMC package BEAST v1.6.1 [57] using an uncorrelated log-normal relaxed clock [58] and the SRD06 substitution model [59], which separates the evolutionary model for the third codon position from the first two. The CD tree was generated from 180–183 sites and the CSD tree from 162–168 sites (Figure S6A, S6B). MCMC Chains ran until inspection of the traces and effective sample size of each parameter using the Tracer program (http://tree.bio.ed.ac.uk/software/tracer) indicated acceptable mixing (ESS>200 for every parameter) and stationarity (as evaluated by the independent runs). For the CD phylogeny, we observed acceptable mixing after a single run of 10 million iterations. The CSD phylogeny required combining three independent runs of 10 million generations each. The first 10% of each MCMC run was discarded as burn-in. Finally, we constructed maximum-clade credibility trees from the posterior tree samples. All analyses were repeated at least once and the results compared for consistency. Evidence of independent evolutionary trajectories of CDs and CSDs (see Results), in addition to the abundance of CD- and CSD- only paralogs, motivated the construction of separate trees for each domain.

### Expression analyses

To investigate expression profiles of each HP1 gene in adult tissues, we extracted RNA from whole bodies, heads, reproductive tracts, and the remaining carcasses of male and female *D. melanogaster*, *D. yakuba*, *D. willistoni*, *D. pseudobscura*, and *D. virilis* using the TRIzol reagent (Invitrogen). Following a DNase treatment (Ambion) and RNeasy (Qiagen) total mRNA clean-up, we generated cDNA (SuperScript III, Invitrogen). A PCR master mix for each primer pair (primer sequences listed in Table S2) was aliquoted into eight tubes containing genomic DNA (positive control), water (negative control), or one of the six tissue-restricted cDNA templates per species. We amplified the housekeeping gene *Ribosomal protein L32* (*rp49*) transcript using intron-spanning primers from all templates in all species to confirm that qualitative comparisons across tissue types for HP1-like genes were robust and to rule out the presence of genomic DNA contamination.

### Population genetic parameter estimates and tests of selection

For HP1 genes that occur in *D. melanogaster*, we estimated several population genetic parameters and ran tests of selection using publically available population genomic data and genome sequences from closely related species. We analyzed 44 alleles parsed from Drosophila Population Genomic Project (DPGP, www.dpgp.org). We treated as missing data all bases with a quality score less than 30, all regions that appeared as identical by descent (IBD), and all regions that exhibited residual heterozygosity (according to the description on DPGP website). We also excluded two alleles of *HP1Lcsd* from *D. melanogaster* that had premature stop codons that shortened the coding region by one codon. We used *D. simulans* polymorphism data from [60] and *D. yakuba* and *D. erecta* alleles from [2] as outgroups. For the population genetic analyses, we only considered sites with at least 20 *D. melanogaster* alleles and three *D. simulans* alleles.

To estimate sequence variation, we calculated π as average pairwise differences [61]. To estimate codon usage bias, we calculated the “Effective Number of Codons” [62] or “ENC” in DNAsp v.5 [63] for single alleles from *D. melanogaster*, *D. simulans*, and *D. yakuba*. We investigated heterogeneous rates of evolution by estimating linage-specific divergence on the branch leading to *D. melanogaster* and *D. simulans* using *D. yakuba* (or *D. erecta* for *HP1Mcsd/Ska*) as outgroup (PAML v.4 [64]). We ranked estimates relative to whole-genome estimates found in [33]. Finally, to test for evidence of positive selection using these population genomic data, we performed a McDonald-Kreitman test (“MK test” [65]).

For the test of selection using a phylogenetic approach, we accessed sequence data from *D. melanogaster*, *D. simulans*, *D. sechellia*, *D. yakuba*, and *D. erecta* orthologs from www.flybase.org. Preliminary sequence data from *D. ficusphila*, *D. elegans*, *D. takahashii* and *D. biarmipes* were obtained from Baylor College of Medicine Human Genome Sequencing Center Drosophila modENCODE project site (http://www.hgsc.bcm.tmc.edu). We aligned orthologous genes in CLUSTALX [66] and fit our multiple alignments to an NSsites model implemented in PAML version 4 [64]. Using a likelihood ratio test to determine significance, we compared models M7 (dN/dS values fit a beta distribution) and M8 (model 7 parameters plus one: dN/dS>1) assuming the f61 model of codon frequencies and multiple starting values of dN/dS. Tree topology was consistent with a previous report [67].

## Supporting Information

Figure S1 Alignment of the *HP1E* syntenic region between *D. melanogaster* (“mel”) and *D. pseudoobscura* (“pse”). The *D. melanogaster* HP1E coding sequence is highlighted in yellow. *D. pseudoobscura* harbors pseudogenized remnants of *HP1E* flanked by conserved regions.
(PDF)

Figure S2 Alignment of the *HP1D2* syntenic region of *D. melanogaster* (“mel”) and *D. simulans* (“sim”). The *D. simulans HP1D2* coding sequence is highlighted in yellow. The *D. melanogaster* region harbors conserved regions flanking the *HP1D2* gene in *D. simulans*, but no evidence of an HP1D2 coding sequence.
(PDF)

Figure S3 *HP1E* syntenic region in the obscura group. (a) Alignment of the *HP1E* syntenic region with the *D. bifasciata* and *D. guanche HP1E* coding sequence highlighted in yellow. (b) Protein alignment of *HP1E* from *D. guanche* and *D. bifasciata*.
(PDF)

Figure S4 Protein alignment of HP1J from *D. virilis* and *D. grimshawii*. The residue annotated as a stop codon in the consensus genome sequence of *D. grimshawii* is highlighted in yellow.
(PDF)

Figure S5 Phylogenetic trees with all support values reported (a) chromodomain (b) chromoshadow domain.
(PDF)

Figure S6 Amino acid alignments for the (a) chromodomain (b) chromoshadow domain.
(PDF)

Table S1 Gene names and symbols of all current HP1 family members. For those genes not annotated, the predicted coding sequences appears under “NOTanno.”
(XLS)

Table S2 Primer Sequences for RTPCR analysis and *HP1E* region sequencing from the obscura group.
(XLS)

Table S3 E-values from new HP1 best tBLASTn hit to *D. melanogaster* genome versus first non-HP1 hit.
(XLS)
