# Supplementary material for: Phylogenomic Analysis Reveals Dynamic Evolutionary History of the Drosophila Heterochromatin Protein 1 (HP1) Gene Family
Source: PLoS Genet. 2012 Jun 21;8(6):e1002729. doi: 10.1371/journal.pgen.1002729 (PMC3380853; doi:10.1371/journal.pgen.1002729)
Supplement: Figure S1 — Alignment of the HP1E syntenic region between D. melanogaster (“mel”) and D. pseudoobscura (“pse”). The D. melanogaster HP1E coding sequence is highlighted in yellow. D. pseudoobscura harbors pseudogenized remnants of HP1E flanked by conserved regions. (PDF) [file pgen.1002729.s001.pdf]

Figure S1.

```
pse  GGATTCGACTGATTTATTCCGTTCACGTACCGCTCACTGCACACTTTGATTGCCGCATTG 926
mel  TAAGTCGCCTGATTTATTCCGGCCACGTAGCACTCACTGCACAGTTTGATTGCCACATTC 857
      .* ***.*************  ****** *.*********** **********.****

pse  GGATGTAAATCTCGGCCTCTAAAAAGAGACCCAACCATTCGGGCCAATCCGCCAAAATCT 986
mel  GCATGTAAATGCTGGCCTCTAAAAAGTATTCAAACCAAGCGACCCAATCC-CACAAATCT 916
     * ********   *************:.: *.*****: **. ******* *..******

pse  GGCCCACGTCAACACGCATAATCGGGCTTTTAATTTCGGGCAATAAAACCTTGGATGCCT 1046
mel  GTCCCACGTCATCGCGCATAATCGAGCTTTTAATTTCGGGTAATAAAACTTTG-----CC 971
     * *********:*.**********.*************** ******** ***     *

pse  TATTTAATGTCACAAACGTGGAAAAACTCTTCTCTCCAGGATT-----------TGACAC 1095
mel  GATTTAATATCAGGTACGTGGCGCCTTCATTCTACAAAAGAAAGCCATAAAATGTGGAAC 1031
      *******.*** .:******....:  .****. ..*.**::            **..**

pse  AAAGTTTGATGAAGAGCATAAAAATAGGTGTTTTTTCAAGTTCTCAAAGAAAGAAACTCC 1155
mel  TGACTTCGAGGGAAGCCATAAACTTA--TGTTTTTCAATGAAGGATAAATAAGAAAAATA 1089
     :.* ** ** *.*.. ******.:**  ******* .*:*::  .:**.:******.: .

pse  GAAAATATTTGTACTAATTAAGAATTCTAT--TACTGAACAAACATACGATAAAATTGGT 1213
mel  ACTAGTTGTTACACCTTTCAAATTTTTAAAGCATATTCCCCAATTTATTGTCAAGTGATT 1149
     ..:*.*: **. ** ::* **.::** :*:  ::.* ..*.** :**  .*.**.* . *

pse  GAAAATAATTGTCGGGAATCCCCATCCGTT--------AAACACAACTTTTCCATCATTA 1265
mel  CATCAACGATTTCTGTGGCCTGCAAAGTTTGCCTTGAAAGGCAAAAGATTTTGTTCATTA 1209
      *:.*:..:* ** * .. *  **:.  **        *..**.** :***  :******

pse  AACATTATGATATTCAAGCCCCACCT-CAACAAACTTTCATCCACGAGTATCCCGAAACT 1324
mel  AACAACGTGATACACGAAACCAAACTGAAAAAAACTTTGTTCTACGAGTATTTC-AAACT 1268
     ****: .***** :*.*..**.*.** .**.******* :** ********  * *****

pse  TTTGGCAAAGTTTACGCATAATTTTTAACGATATTTTCCCGCATTTAGCCTTCGAGTGG- 1383
mel  TTTCGCCAAGTTTACGCATAATTTTTTACGATATTTTCCGCAGTTTTTCCTTTTGACGGC 1328
     *** **.******************:***********  ..***: ****  .. **

pse  -----TGGTTGCTGGTGTTGTCTTCGAGCACTTTTGTTGGCCTCCACGGATTCCAAATAC 1438
mel  TCATCTTATTCTTATTGTTGTTCGAAAGGACTTACCCAG-CATCCAGGGATCTCTAATAG 1387
          * .**  *. ******   ..** ****:   :* *.**** ****  *:****

pse  GTTTCCGGCCAAACAACTTTATTCAATTTCTCATATTGCATTGGCATTGGTATTTTATTA 1498
mel  ATTTCCGG-CAAACAATTTTATTCACTTTCTGCTATTGAATT-----TGGTATTAT-TAA 1440
     .******* ******* ********.***** .*****.***     *******:* *:*

pse  AGCTGTTATGCTCTGAGCTCCTGGATATTAAGATAAGACAACTTCCTGGTAAGTTTTCTT 1558
mel  TGCCGCATTTC-CTAAGCTGTGGGCATTAAGTATATGGCTAACTTTT--------CTACT 1491
     :** * ::* * **.****   **.::*:*. ***:*.*:*. *  *         *. *

pse  CCTGAATATTAAATAAATTTCGAGTGTTTAGTATTAAAATTTTATGGTAATCTCCGGCAA 1618
mel  TCAGGATATTAAACGAATTCATTACAATATTTTTCATAATTTTAT--------------- 1536
      *:*.******** .**** . :. .:*:: *:* *:********

pse  ACGATTGATATTTCTTTGCCAAACAAAATTTGGATGGTTTTTATTTTCGCAAAAAATTAA 1678
mel  ----------TTCTTTTGGCAAGAAGATATTAAAG------TAGACCAGCATTTAAAAGA 1580
               **  **** ***..*.*::**..*      ** :  .***:::**::.*

pse  AATATTGCTCCCGTAAAATAGATGACTTATTGATGCAGTAGCAATGGTATAATTACAATT 1738
mel  AAACTTG--TCTGCACAGAAAACTACGTTTTGTTG-AGTAGCAACTGTAGATAAAAGTT 1637
     **:.***   * * *.*.:*.*  ** *:***:** ********  *** *:::*.*.**

pse  ACATAATTACAAAGGCAATTTGCTTGTGCCTTTTTTAAAAATTATTGTATTTTCCTAGCC 1798
mel  ACG------AAAATGTTTTTCGCTCTG-----------ACATTGCTGTCAAAACTCATCA 1680
     **.      .*** * ::** ***           *.***. ***.::::*  * *.

pse  TAAAATTTTCCTCGTAAACAACATAGGGCTGCAAACGATTTGGTTATCGGAATGGAAAAA 1858
mel  AAAAAATACTATTTGAACATTTAAAAATATAAAATGGATTCAGATGCCG--ATGGTGGCG 1738
     :****:*:  .*   **..:: *:*.. .*..**: **** .*:*. **  ****:....

pse  AAATCACTTTGTGCGCGTAACAGCCCAATCCAACAAAATAGTTGAAAAAAGTTGACCGTG 1918
mel  AGACCGTTT----CGAATTTCT--------CTGAAAATTCGACGGATTTTGAAGAAACTG 1786
     *.* *. **    **..*::*:        *:..***:*.*: *.*::::*::**.. **

pse  GAAAGTGTGTCAATGTCAAACCAAAAGGAAGACGACAAAAATTGACAAGCCATAAAGGTA 1978
mel  AGGAGTATATTGTAGAGAGGATTTTGGATCGAAGGCATTATATG---GGCCAAT----TA 1839
     ...***.*.* .::*: *... :::.*.:.**.*.**::*::**   .****::    **

pse  CGATAAAAAGTCAAGAGAAAATATTCCAATGACTTTCGAACAAATTTATTCCATTGGCCA 2038
mel  CAATACTTGGTCAAATGGCTGGATTATAG-------CGATGAAGATAATACCTGGG---- 1888
     *.***.::.*****.:*..:. ***. *.       ***: **.:*:**:**:  *

pse  ACACGATTGTAGTCCATAAATATTCATGGTAAATTGAACTATTGAATGTTTTGTTGTACC 2098
mel  AATCGGCAGCAGATCTCG-------------------------------ATTGCCATTCA 1917
     *.:**. :* **: *: .                               :***  .*:*.

pse  CCAACTGCTGCATCTGGAAATCATTAGAAGCATGGGAAAATCGGATGAAGCGAAGGCCCA 2158
mel  CTTATTGAT----------TCGATTGAATCACAAAAAGTCTGAAGAGG---------- 1956
     * :* **.*           **.:*:*** ** ...***.** **:**.*

pse  TTCTAGTTCCTTATCGGAGATGAGACTTATCTTCAACACAGCATTAAAGAAACCAACCAA 2218
mel  ---------------GGTCAAGAGCTGAATAATCAATATGAAACTAAAG--------CTA 1993
                    **: *:***.  :**.:**** * ...* *****        *:*

pse  AACGTACGATTGAAAGTTGATCCCGCTGTAGTAATTGATTATCCTTTCAGTCGTGGTTTC 2278
mel  AACG----ACTGAAAATCGATCCATGTCTAGTAGTAGATAGTCCTTTTAATCATGGTTTC 2049
     ****    * *****.* *****.  * *****.*:***:.****** *.**.*******

pse  AAAGCTGAACAGATGCTCGAAGGTTTCAAAAAGGGAGAACAAATATCGCTTCTGATCAGG 2338
mel  ACTGCACAAGAGATTCTAAAAGGCGCTAAAAACAATGGTGAAATATCGTTTTTAATTAGG 2109
     *.:**: ** **** **..****    ***** ..:*.: ******** ** *.** ***

pse  TCTATGTCTGTATCTAGGCTTCGCCTATGTGGAAATACCCCACACGGTCTTCAAGTTCTA 2398
mel  ------------TTTTGGCATC-------TCGATCAACCACAAGTGGTG----------C 2140
                 * *:***:**       * **:.:***.**.. ***           .

pse  CGAAAAGAATTGCCAATTTGCAAAACTTAATAAGGATTGAATGAAAAACAAGACATTGTG 2458
mel  CAAGTGGAATTGCATATGTGGAAATACCGCAGATGGTT--CTGAAATTCTATGAAAATCA 2198
     *.*.:.*******.:** ** ***:.  ..:.* *.**  .*****::*:* ..*::  .

pse  TTGGACGTTTTGGGCACTTTTTTCGTCAAAATTGCCTTAAGATTCTTAGCAGATAAATAA 2518
mel  TTG--CAATTTTCACGATTATTTAAATAATTAT---ATATCATGACTTGCAATTCTGGAT 2253
     ***  *.:***  .*..**:***..: **:::*   :**: ** . *:***.:*.:. *:

pse  CACCAAATCCTAATATGTATTATATTAATTTTTATTCTTATTCCATTAAACTGTTGTACT 2578
mel  CTTCAAAAAAAAAAAACTTATGAATATGTGTGAGCTTCTTATATT------TTATATG 2307
     *: ****:..:**:*: *::*.:**::.* * *.: ***.**. ***        **.**

pse  GCAGTTCCATTTGTTGTTTCTTTTATTTTACACATATTTGCACCATTAAATTGTGCCACT 2638
mel  TTAGGTTAAATTGCTTTACTTTTTATAATAAAAG---GTGTCCCTT-----TGCACAGCA 2359
       ** * .*:*** * *:  ******::**.*..    ** .**:*      ** .*..*:

pse  TTACGCAACATAATAAAAGGAAAAATGCCAACTTTTAAGTGATTTTCGAGGCGTGTTTGT 2698
mel  TTACAAACAATAATGAAGG-CAAAATGCCAAGTTTTAAGTGATTTTTGGGGCGTGTTTG- 2417
     ****..*..*****.**.* .********** ************** *.**********

pse  TCCGGGTGCCCCCGAGTAAACATTCCTAATTGCAACAACAACATCG---ACTATGGGGCA 2755
mel  CCCGACAGGCAGCG--TAAACACTCTTAATTGCAACAACAGTGCCGGCAAACAAGGGCCA 2475
      ***. :* *. **  ****** ** **************. . **   *. *:*** **

pse  G------GCACCAAACAGAAGAGGCTCGAGGAATTTACAGCACGGGAAAATGAAGACAAA 2809
mel  GGAATGTACGACGGCCAAAATGAAGCGAAAATATTTGCATTCCGTTTTTGTGGCCATTCC 2535
     *      .*..*...**.** ...   .*..:****.**  .**  :::.**.. * :..

pse  ATATTTGCATTCCTTTTTTTTTTGTTTCTCT---CTTTTGGCCCCCCGTGGTGGATGGGG 2866
mel  CTTGTCTCATCGCAAGTACCAAAAGTCGTAAGACCTATTCTCTGTTTTTGGCTGATGGGG 2595
     .*: *  ***  *:: *:  :::. *  *.:    **:**  *      ***  *******

pse  CGTG---CCTGGTCGGCCAGTGGCC---TATATTTGAGTCCGTTATTCAGCGTTTTGGTT 2920
mel  CGTGGTGGCCGGACGTACTTTGCCCTATATATTCGTGTTTGTTTTTCGTCACTTGGGTC 2655
     ****    * **:** .*: * ***    ****** *:**  ***:***. *. ** ***

pse  TGGTTTGTTG-CGTTACTCCCAACAGGCACGTAGTAGCACCAGCAGCCGCCATAGGGAAT 2979
mel  CTTTTTTTTGGAGGGGGGGGGGGGGGGCTCTGGTGCTACCCAGTTGCGTCCAGTGGGCAC 2715
        *** *** .*  .     .. .***:*  .  . ..**** :**  *** :***.*

pse  GCTTTGTTTATG-CAGCAAGGAA-TTCCAAATGCAGTTCCTGCAGTTCCACCAGAATGGA 3037
mel  GTAGCCACAATAGCAGCAAAAAGGTTTCGTTTAAGCCTTCTGCACTTTTGCCG------- 2768
     * :   : :**. ******..*. ** *.::*...  * ***** **  .**.

pse  AAACTTTGATACTGCAGTTCTATATATAGAAAAAATGTGTACTG-CAGTGGC-CATATAT 3095
mel  -AGAATTGATACTGCAATATCCGGTCGAGCAAGTTCAACTCCAATCAATTGCTCTTCGAT 2827
      *..:***********.*:  . .*. **.**.::.: *.*:. **.* ** *:*. **

pse  GTAGAAAAGTGTTT---AGCTTACAATATATAGAAGCCCGCTTTCAATTAGTCAGACGTC 3152
mel  ATTGGTAAGTTTTTGTCGGCGGTAAAAGAAACTAAGACCTATTTGAATTTATAGGCACAC 2887
     .*:*.:**** ***   .**  :.**:.:*:. ***.** .*** ****:.*..*.. :*

pse  TGATCTTTTGTGACTTGATTTTTGTAGATTATAAGGGTATGATAATATATGTATGTCACA 3212
mel  GCTTGATTC--AACTTTTGTTTTGACGTTATGCTTGCACTGGTGTTCAAT-ACTATTTCT 2944
      :* :**   .**** : *****:.*:*:: .: * :.**.*.:*.:** :.*.* :*:

pse  GGCAAAAGAAAACATACATTGTACTC-----TTGATTCCAGATTCCTGATCAGCATCAG- 3266
mel  ATTCAAAATATTCTTAGATTAAGCTAGATAACTGATCCATGAAACTAGTGAAGCATTTAT 3004
     .  .***.:*::*:** ***.:.**.      **** *.:**::* :*: .***** :.

pse  CCACCCTATTCAGTCGTAACATCAGCCATAGCACCCTTACTCCGTTTCTTTTTAAGGGTA 3326
mel  TCACGCTGTGCTTTTTAGATTTTGTGAGTTAAAAAGAATAATGTTTTTTTCGATTTT 3064
      *** **.* *: *  **..:* :*  * : .*...: *.*..  ** *****...  *:

pse  ATCAAGCGTCGCTTTCATGAACAAAACAG-AAAAAAAAAATTAATTGCGTGCCCAGGCGT 3385
mel  CACAGAAATAATTGAAAAAAAGGAGGGGGAAACAAAAAATTAATTTGGCGCCTA--CAT 3122
     .:**....*.. * :.*:.** .*.. .* ***.***********  * *** *  *.*

pse  TGGCTTTTCCTCCTTTCCCATCCTGCCACATGCCATTATTTTTTGTTTTCCATGAGGTCA 3445
mel  T--CTATTGCCCACATTCCAACCT------ATCCACTTTCTTTTGTTTCGCATGAGGTCA 3174
     *  **:** * *. :* ***:***      : *** *:* ********  *********

pse  AGGGTTAAGTACATACACACATGCAGTATTTGTTTACGTGCTTGAGCCGAACCTCAAACA 3505
mel  AGGGTTAAATGTACACACGCATACA-----TACATACATGCATGATGACCGTATTTGTTT 3229
     ********.*. * ****.***.**     *. :***.***:***  . .. .* :.: :

pse  ATGAACGACCCACACCCATTGTTCCAAGATGTAGAGTATGCTCTATGAATAGCAGAGTTT 3565
```
