# Supplementary material for: Phylogenomic Analysis Reveals Dynamic Evolutionary History of the Drosophila Heterochromatin Protein 1 (HP1) Gene Family
Source: PLoS Genet. 2012 Jun 21;8(6):e1002729. doi: 10.1371/journal.pgen.1002729 (PMC3380853; doi:10.1371/journal.pgen.1002729)
Supplement: Figure S2 — Alignment of the HP1D2 syntenic region of D. melanogaster (“mel”) and D. simulans (“sim”). The D. simulans HP1D2 coding sequence is highlighted in yellow. The D. melanogaster region harbors conserved regions flanking the HP1D2 gene in D. simulans, but no evidence of an HP1D2 coding sequence. (PDF) [file pgen.1002729.s002.pdf]

Figure S2.

```
sim             CCTTTCCAACGTCCGTTTTGTTCAAAGTTCGCTTTTCGACTGACCAGACTCGGCTTTCGG 60
mel             ----------GTCCGTTTTGTTCAAAGTTCGCTTTTCAACTGACCAGATTCGGCTTTCGG 50
                          ***************************.********** ***********

sim             TTGCGCGGCCAAAAATGCCAAATGCCGCGCACAGTGTGACCATCCGCCAGTGAGGCGTAA 120
mel             TTGCGCGGCCAAAAATGCCAAATGCCGCGCATAGTGTGACCATCCGTCAGTGCAGCGTGA 110
                ****************************** ************** *****..****.*

sim             ATACCAAAAAGCTCACGCGCGGATG-TTCGGTTACACTACAACAAAAGCTTTTGGTAAAA 179
mel             ATACCGAAAAGCTCACGCGCTGATGCTCCGGTTACACTACAACAAAAGCTTTTGGTACAA 170
                *****.************** **** * ***************************.**

sim             ATCGAGGCT---AACGGCTTTTTAAAACGCAATCTTCATTTAATATGTTAAGCATTTATT 236
mel             ATCGAAGCTTACAACAGCTTTTTAAAACGCAATGAACAAAATATTTACAGAGTATT---- 226
                *****.***   ***.***************** ::**::::**:*. :.** ***

sim             GAAAAATAAATAATTGTCGCATTACAATGCAACCTTGGCAACCAGACTATATTTGAAATG 296
mel             ----------------------TACACGACGAGCTT-----------TTTTTTTCAATTG 253
                                      ****. .*.* ***           *:*:*** **:**

sim             GACTTGGGCAAAACATTATGGTGTGGGGGAGGGGGGAGGGGGAGGCAGAAACAGCACGAG 356
mel             -------TCAACTAATTG----------------------------AAAAAAAGCTCGT- 277
                        ***.:.***.                            *.***.***:**:

sim             CGTCCGTTTGAAAAAGAAACATCAAATTCGCAGAAGGAAGGAAGAAATAAAAAATAAGAA 416
mel             ------TGTGTAAAT-ATTTTTGACAACCTCTGTG------------------------- 305
                      * **:***: *:: :* *.*: * *:*:.

sim             AAGACTTCTTTCTTCAAATATTCAGCTATATTCTTAAATTTCACCTTTACTGGCTAAATA 476
mel             ------TATATATACATATGTACATATTTAATAT---GTTACACATTTTTG------AA 350
                      *.*:*.*:**:**.*:** .*:**:*.*   .**:***.***: **      :*

sim             ATTTCATTAAATATCTAGTGAAATCTTATTTAAAGATGCGCATGATCTTACCAATTAGTT 536
mel             AAATAAATAATTG------------------------GCGCAATACAGGGCCACTTTGGT 386
                *::*.*:***:*.                        *****: * .  .***.**:* *

sim             TTTTATGCGGAAAAAGCGAAAAGAAAGTTAAAAGTTTTAAAATAAAATCATGATTCTCAC 596
mel             TTACAT------------------------------------------------------ 392
                **: **

sim             TCCATTCCAAAAAAAACAAACTTTTGTGAAAATTGCCTAATTTTGGAATAGTAGTGGAAC 656
mel             ------------------------------------------------------------

sim             AAGCACGTGCGCCACATTCTTTCGTGTTGGCAACGTCATTACGGTCGCAGGAGTTTCCTC 716
mel             -AGCACAGGCAAAACAT--------GTTG------------------------------- 412
                 *****. **...****        ****

sim             ATTTCTTTTGATTCGGCAACGAAAATATTAAGTAAAATGCGCAACCCACAGCGGAGAAAC 776
mel             ----------------CTTCGGAGATTCTAA--------------------------TAC 430
                                *::**.*.**: ***                          :**

sim             ACGGTGGACAGCCAGAAGTATGTGGTGGAGAAGATCGTTGGAAAGCGCATCGTAAAAGGT 836
mel             ACG-------------------------ATATAAACGT---------------------- 443
                ***                         * *:.*:***

sim             CGTCTGCAGTTTCGTGTCAAGTGGATGCACTATCCGATGAAAGCGAGCACATGGGAGCCA 896
mel             ------------------------------------ATGAAAACAAGCA----------- 456
                                                    ******.*.****

sim             GTGGAAGCATTGGGCCACCTCTCGCGCTTGCTGGCCGATTATGAGGCGAGGCAGTACAAG 956
mel             -------------------------------TGAACGAATATAAAGT------------- 472
                                               **..***:***.*.*

sim             CGCTTCCGGAAGAAGCTGGCCAAGGCGGCAAAGGGCCCCAAATCGCGAGCCAAAAGCAAG 1016
mel             CCCTTCGG-------CTTTCCAAGCCTGCTGG---------------------------- 497
                * **** *       **  ***** * **:..

sim             GCTAAGAGCGCCGCCCGTCGACGCCAATCGCGCCACGTGCCCAGTGGCCAAGCAGCAACA 1076
mel             ----------------------------------------------------------AT 499
                                                                          .:

sim             TCGGCTACATCGGGCTCGGCCGCAGCGGGCCAAGGAGCAAATGGGGAACGCGGCAGTAAT 1136
mel             TTATCTTCATCGG---AGGTTTCATCTG----AGGTG-ATATGG---ACGAAGATGGAAT 548
                * . **:******   .**   ** * *    ***:* *:****   ***..*.:* ***

sim             GAGCAATCATCAGGGAGTTCGGTGGTAGCTCGAGGAGTTGGTCTCGCGCATGAGCGCAAG 1196
mel             GA----------------------------CGAG-------------------------G 555
                **                            ****                         *

sim             CGTGTCGATCCGCTGGCTGTGGAGCAGCCAAGGATCGAGGGCGTGCAGCATCCACGCACT 1256
mel             CATTTTTTTCCGAT---------------------------------------------- 569
                *.* *  :****.*

sim             TCACGCGGCACATCCGCGCAGGTACCGAACTGGAAAATGGAGCCGTACAGGGGGTCCTTC 1316
mel             -----------TTACACGCAG--------------------------------------- 579
                           :*.*.*****

sim             GGTTTGGCGCGTGGCCTGGAGCTGGAAAAGGTGCACCACTGCTTCTTTGTTCGCAGGCAG 1376
mel             ------------------------------------------------------AAGCAG 585
                                                                      *.****

sim             CTCTTCATGTTCGTCACCTGGGTGGACACCCCACGATGGACGCGGTGCTGCTTAGCGAC 1436
mel             CACG-------------------------------------------------------- 589
                *:*

sim             ATACGACACGCATATCCGATGCCAATAATTAAATACTTTCAAAATTTGGCCTTCCCAAAT 1496
mel             ------------------------------------------------------------

sim             TACAAAGATGCCTGAATTCAAGGGAATCGCATTTCAAACATAGATTAAGCGTTTTTTTTT 1556
mel             -----------------------------------------------AGCGTCCTTTTT- 601
                                                               *****  *****

sim             TAACTCCATCTAGATAGCATAGCATTTTAACATGTTTGTACGCAGCTCGATCGTCACTAT 1616
mel             ------------------ATAGCATTTTAATATGATTGTACACAGTTCGATCGTCACTAT 643
                                  ************ ***:******.*** **************

sim             GCGATTGTTTAATCACTAGTCAAGCTTTCCACGACATGTGACCAGATTTCAGTCGCCTTT 1676
mel             GCGATTGTTTAATCATTATTTAAGCTTTTCAAGACATGTGACCAGATTTTAGTCGCCTTT 703
                *************** ** * ******* **.***************** **********

sim             CCAGCCGAGCTTCCGAAAATCTGATACCTTATACTATGGCGCCATTCGCGCAGCCT--CA 1734
mel             CAAGCTGAGATTCCGAAAATCAGATACTTTATACTATGGCGCCATTCGCGCAGCCTCACA 763
                *.*** ***.***********:***** ****************************  **

sim             CTGATTGTAAACAAGTAAGCGGATACGCTGTTAGCCAAATCATAAACACCAGCGATCAAT 1794
mel             CTGATTGAAAACAAGTAAGCTGATAG-CTGG----------ATACGCTGCAAGGCAAAGT 812
                *******:************ ****  ***           ***..*: **. *.:.*.*
```

```
sim  CCTTTCCAACGTCCGTTTTGTTCAAAGTTCGCTTTTCGACTGACCAGACTCGGCTTTCGG 60
ere  CCTTACCAACGTCCGTCTTGTTCAAAGTTCACTTTTCGACTGACCAGACTCGGCATTCGG 60
     ****:*********** *************.************************:*****

sim  TTGCGCGGCCAAAAATGCCAAATGCCGCGCACAGTGTGACCATCCGCCAGTGAGGCGTAA 120
ere  TTGCGCGGCCAAAAATACCAAAAGCCGAGCGCAGTGTGACCGTTCGCCGGTGCAGCGTAA 120
     ****************.*****:****.**.**********.* ****.***..******

sim  ATACCAAAAAGCTCACGCGCGGATG-TTCGGTTACACTACAACAAAAGCTTTT-GGTAAA 178
ere  ATACCAAAAAGCTCGCACGCTGCTGTTACCGTCACACTACAGCGAAAGCTTTTTGGTAAC 180
     **************.*.*** *.** *:* ** ********.*.********* *****.

sim  AATCGAGGCTAACGGCTTTTTAAAACGCAATCTTCAT----------------------- 215
ere  ATTCAAAATAAAGAGCTTTTTAAAACTTTTTTTAATTAACTTAATTTGTTTTTTTAACT 240
     *:**.*.. :** .************  ::* **.**

sim  ------TTAATATGTTAAGCATTTATTGAAAAATAAATAATTGTCGCATT---------- 259
ere  AAATTATTAATATGTTCTGCGTTTTTTTTTAAATATATATTTCTCGCATTTGGTTTACAC 300
           **********.:**.***:** ::*****:***:** *******

sim  ------------------------------------------------------------
ere  ACGGTACGCCGGTGCAGCGCACATTCCAAAAAGCTCTCACGCTGCTGTTAGCGTCGCACT 360

sim  ------------------ACAATGCAACCTTGGCAAC-------CAGACTATATTTGAA 293
ere  ACAGCGAAAGCTTTTTGTTAACATTCAAAATTAACAGCGTTTTGAAACACAATTAGCTAC 420
                       *..** ***..**..**.*       .* **:**::   *.

sim  ATGGACTTGGGCAAAACATTATGGTGTG-------------------------------- 321
ere  ATTTACTTTGTAACTAAATTATCATATGTTCTGCATTTTTAAGAAATATATATTTCTCG 480
     **  **** * .*.:*.***** .*.**

sim  -----GGGGAGGGGGGAGGGGGAG-------GCAGAAACAGCACG--------AGCG--- 358
ere  CATTTGGTTTATACATAAGTAAAGTACATAAGCACATACATAAAATGAATTTATGCGGTG 540
          **  :. . . *.* ..**       *** *:*** .*..        :***

sim  -----TCCGTT----------TGAAAAAGAAACATCAAATTCGCAG----------AAGG 393
ere  TTTGGAACGTTGTCAATAACATGAACATGAAAACACAAATTCGCTTGCATAGACAACAGG 600
          :.****          ****.*:****..:*********:          .***

sim  AAGGAAGAAATAAAAAAATAAGAA-------------------------AAGACTTCTTTC 428
ere  AAAAAATATATACATATATATAATACTATTTAGGCCCATCGGCGCTCCAAGCCTTAATCC 660
     **..** *:***.*:*:::* **                          ***.***.:* *

sim  TTCAA------------------------------------------------------- 433
ere  TTCAGGCTGGTCACCGAGCTGGTTGAAGAGCCACTGGACGCGCGGGCAATACTGATGCTA 720
     ****.

sim  -------------------------ATATTCAGCTATATTCTTAAATTTCACCTTTACTG 468
ere  CCTCAGACGTAGTGACCCATTTCTGATCCTCCGCCGTGATCAATGGTTTCGCCTCATCTT 780
                              **. **.** .*.:**:::..****.*** ::**

sim  GCTAAATAATT-------------------TCATTAAATATCTAGTG------------A 497
ere  CTTATGTCCTGGTAGTCCACCCCTTAGGTGCCGCTGCTTGTCTTGCTGCTCCTTCTCCAA 840
       **:.*..*                   *. *..:*.***:*               *

sim  AATCTTATTTAAAGATGCGCATG----------ATCTTACCAATTAG----TTTTTTATG 543
ere  GGTCTTCATGTCGGACTCGTCTGGGTTCGTTTTCTCCAACTGCTCCGGCTTCTTATTCTG 900
   ..****.:* :..**  ** .**           .** :** ..* .*     **:**.**
```

```
CGGAAAAAGCGAAAAG----------------------AAAGTTAAAAGTTTTAAAATAA 581
CTGGGACCGCTTGTAGCTCTTCCGCTCGTATTCGCCCACCAGTTTGCAGCAGTTAGACAT 960
* *..*..** :.:**                      ..****:..** : *:*.* *:

AATCATGATT--CTCACT-----------CCAT--------------------------- 601
GTTCTCGTATGGCTCCCAGGTGGCCTCGACCATGGGAAAGCCCAGCCACTTGACAAAGTA 1020
.:**: *::*  ***.*:           ****

---------TCCAAAAAAAACAAACTTT-------------------------------- 620
CTGAGCGCGCCCGTTCTGAACACGCTTTGCGAGGATCTTCTCCACTTCCCATTCACCATT 1080
         **.::.:.****..****

-----------------------------------------------------------T 621
ATGGCCATTTGGGAGCGATGCATCGTCCTCGGAGGCATCATCTGATGTGATGGGGATGGT 1140
                                                           *

GTGAAAATTGCCT-------------AATTTTGG-------------------------- 642
GTTGAATTTACGAGACATCTTGTTAAATTTATGGGGAAAAATCACAATTCTTTTGAAAAC 1200
** .**:**.* :             *:**:***

----------------------AATAGTAGTGGAACAAGCACGTGCG-------CCACA 672
GAAATCAAACGACTTCACAGGAAAACGGAAATGAAAAATTCACTTGTCGCTTCTTCCTCA 1260
                      ** .*:*.**.**.*: *** **         **:**

TTCTTTCGTGTTGGCAACGTCATTACGG------TCGCAGG---------------AGTT 711
AACTGCCAACTCGCCGAAATCTGCAAGGGCTGCATAACAAGTTTAGGCACTGTTGCGGTT 1320
::**  *.: * * *.*..**:  *.**      *..**.*               .***

TCCT-------------------------------------CATTTCTTTTG-----AT 728
TCCAGTCACGATATGCAAATAGTCACTTAATTCTTTTCAAAGCATTTTTTTTTTTTAATTT 1380
***:                                     ***** ****      :*

TCGGCAACGAAAATATTAAG--------------TAAAATGCGCA----ACCCACAGCGG 770
ATATATACGTAAATATTAAAGTCTTAAGCCTATTTAAATTATGCATAGAACACACACCGT 1440
: . .:***:*********.              ****:*. ***    **.**** **

AG----------------------AAACACG----------------------------- 779
AGGATCTTGTAAAATAGTATGTTTTTACGCGGATAAAGCGAGCAAAAAAAAAGAAAGTA 1500
**                      ::**.**

-------------------------------------------------GTGG----- 783
TGAAATTAACGTCTTTTAAAATTAACTTAGTTTCTGCAAAAATAAGCATTAGTGGAAAG 1560
                                                  ****

---ACAGCCAGAAGTATGTGG--------------------------------------- 801
ACTGCATTCGGAATTGTAATGGAAACTAGTTAACCTCGAGTGCACTACATTTAAGCGCGT 1620
   .**  *.*** *.*.: *

--------------------------------------------------TGGAGAAGAT 811
TGGCAGCCTTCAAGTTCGCTCGAGTTCTTTTTGTTTTAACTCGACAAAACTGGAAAATAT 1680
                                                  ****.** **

CGTTGGAAAGCGCATCGTAAAAGGTCGTCTGCAGTTTCGTGTCAAGTGGATGCACTATCC 871
CGTTGGAAAGCGCTTCCAGGACGGTCGTCTGCAGTTTCTAGTCAAGTGGCATGGTTTTGG 1740
*************:** :..*.**************** :*********.:  . *:*

GATGAAAGCGAGCACATGGGAGCCAGTGGAAGCATTGGGCCACCTCTCGCGCTTGCTGGC 931
GGAGGAAGAGAACAGCTGGGAGCCAATGGAAGGAGTGGGTCACCTCTCTTACCTGCTGGC 1800
*.:*.***.**.** .*********.****** * **** ********  .* *******
```

```
CGATTATGAGGCGAGGCAGTACAAGCGCTTCCGGAAGAAGCTGGC--------------- 976
CGATTTCGAGGCGCAGGAATACAAGCGCAGCCAGTTGAAGGAGGCGCCGAGGAGTAGCGA 1860
*****: ******..* *.*********: **.*::**** :***

--------------------CAAGGCGGCAAAGGGCCCCAAATCGCGAGC---------- 1006
ACAGTCCAACCGATCCTCCATCAAGGCGACAAAGCGCCGGAATTCCCGAGCGGTTGACTC 1920
                    *******.***** ***  **:** *****

----------------------------CAAAAGCA----------------------- 1014
TGGAAAAGCCAAGAGAGTCGCATCCAATCCTAATGCAGCCAAAGCCGCGATCAGTCCATA 1980
                            *:**:***

-----AGGCTAAGAGCGCCGCCCGTCGACGCCAATCGCGCCACGTGCCCAGTGGCCAAGC 1069
GCCCTTGGCCAAGGGCCAAACGCGTCGACGCCAATAGCGCTATATGCCCACGGACGACTC 2040
     :*** ***.** ...* *************.**** * .******  *.* *. *

AG---------CAACATCGGCTACATCGGGCTCGGCCGCAGCGGGCCAAGGAGCAAATGG 1120
CAGTGGCCATGCACCATCTACTTCATGGAACACAGCTGCAGCGGACCCAGGAGCAACAGC 2100
..         **.**** .**:*** *..*:*.** *******.**.********.:*

GGAACG---------------CGGCAGTAATGAGCAATCATCAGGGAGTTCGGTGGTAGC 1165
TGAGCACAAGAGCTCGTTGAACGGCTGTAAGGAGCAACCATCAGAGAGCTCGGTGGTAGA 2160
 **.*.               ****:**** ****** ******.*** **********.

TCGAGGAGTTGGTCTCGCGCATGAGCGCAAGCGTGTCGATCCGCTGGCTGTGGAGCAGCC 1225
TCATCCAGTTGCTCTCGCGCCGGAACGCATCCATAACTCACCGCTAAATATGGAGCAGCC 2220
**.:  ***** ********. **.****: *.*.:* .:*****...*.**********

AAGGATCGAGGGCGTG------------CAGCATCCACGCACTTCACGCGGCACATCCGC 1273
GCAGGTGGAAGGTGTGGAGCTGCTCCTGCCCCTTCCGGGCACTTCAAGCAGTGCATCTGC 2280
...*.* **.** ***            *. *:***. ********.**.* .**** **

GCAGGTACCGAACTGGAAAATGGAGCCGTACAGGGGGTCCTTCGGTTTGGCGCGTGGCCT 1333
GCAGGAACCGGATTGGAGATTGCCACCGTACAAGAAGCCCTTTGGTTTGGCGCGCGGCCT 2340
*****:****.* ****.*:** ..*******.*..* **** *********** *****

GGAGCTGGAAAAGGTGCACCACTGCTTCTTTGTTCGCAGGCAGCTCTTCATGTTCGTCAC 1393
GGAGCTGAAGAAGGTGCACCACTGCTTCCTTGTTCGCAAGCAGCTCTTTCTGTTCGTCAG 2400
*******.*.***************** *********.********* .*********

CTGGGTGGGACACCCCACGATGGACGCGGTGCTGCTTAGCGACATACGACACGCATATCC 1453
CTGAAAGGGTTGATCCGTGATGGACGCTGTGCTCCTGAGGGACATACAGCATGCATTCCC 2460
***..:***: .. **. ********* ***** ** ** *******..** ****: **

GATGCCAATAATTAAATACTTTCAAAATTTGGCCTTCCCAAATTAC-------------- 1499
AATACCAATAATAAAATACTTTGAGAGTATTTCCGTCGTAAATCACGAGGACCAAGAGGA 2520
.**.********:********* *.*.*:*  ** **  **** **

-------------AAAGATGCCTGAATTCAAGGGAATCGCATTTCAAACATAG-----AT 1541
GAGACGGAACCACAATGTTGCATGAACTCAAAGAGCGCGCATTTCAAACTTGGTAGAAAG 2580
             **:*:***.**** ****.*... ***********:*.*     *

TAAGCGTTTTTTTTTTAACTCCATCTAGATAGCATAGCATTTTAACATGTTTGTACGCAG 1601
TATGCTCAATATTATTGAACTCAACTTGAACTCTTAGCTTTT-AATATGTTTGTACGCAG 2639
**:**  ::*:**:**.*.  **:**:**:. *:****:*** ** **************

CTCGAT-CGTCACTATGCGATTGTTTAATCACTAG--------TCAAGCTTTCCACGACA 1652
CTCTTTGCGGGCGCACACGAATCTATCATCACCAAGCGATTCATTAAGCTTTTCACGACA 2699
*** :* **  .  * .***:* *:*.***** *.         * ******* *******
```
