# Supplementary material for: Phylogenomic Analysis Reveals Dynamic Evolutionary History of the Drosophila Heterochromatin Protein 1 (HP1) Gene Family
Source: PLoS Genet. 2012 Jun 21;8(6):e1002729. doi: 10.1371/journal.pgen.1002729 (PMC3380853; doi:10.1371/journal.pgen.1002729)
Supplement: Figure S3 — HP1E syntenic region in the obscura group. (a) Alignment of the HP1E syntenic region with the D. bifasciata and D. guanche HP1E coding sequence highlighted in yellow. (b) Protein alignment of HP1E from D. guanche and D. bifasciata. (PDF) [file pgen.1002729.s003.pdf]

Figure S3.

(a)

```
D.pseudoobscura    GTCACAAACGTGGAAAAACTCTTCTCTCCAGGATTTGACACAAAGTTT--GATGAAGAG- 57
D.persimils        GTCACAAACGTGGAAAAACTCTTCTCTCCGGGATTTGACACAAAGTTT--GATGGAGAG- 57
D.affinis          GTCAAGAACGTGAAAGAACTCGGCTTTCCGGGATTTGACACAAAGTTTTAGAAGGAAAG- 59
D.azteca           GTCAAGAACGTGAAAAAACTCGGCTCTCCGGGATTCGACACAAAATGTTAGAAGGAGAG- 59
D.bifasciata       GTCACGAACGTGAAAGAACTCTTCTCAGAGGGATTTGACACAAGGTTTC--AAGGAGAGG 58
D.guanche          GTCACGAACGTGAAAAAACTCTTCTTTGCGGGATTTGACAAAAAGGTTT--AAGGAGAGG 58
                   ****  ****** ** *****  **     ***** **** **    *   * * * **

D.pseudoobscura    CA-TAAAAATAGGTGTTTT-------------TTCAAGTTCTCAAAGAAAGAAACTC--- 100
D.persimils        CA-TAAAAATAGGTGTTTG-------------TTCAAGTTCTCAAAGAAAAAAACTC--- 100
D.affinis          CAGTAAAAATGGGTGTTTTC------------TTCAAGTTCTCAATAAA----------- 96
D.azteca           CAGTAAAAGTAGGTGTTTTC------------TTCAAGTTCTCAATAAAGGAAAACCCAA 107
D.bifasciata       CAATCAAAATA----CTTG----------AAATAAAGACACTCCAAAAGT---------- 94
D.guanche          CAATAAAAATAGGTGTTTGCCTCAAGAAGAAATCAAAAAATTACAAAAGG---------- 108
                   ** * *** *      **                *  *    *  *  *

D.pseudoobscura    --------CGAAAATA-TTTGTACTAATTAAGAATTCTATTACTGAAC--AAACATACGA 149
D.persimils        --------CGAAAATA-TTTGTACTAATTAAGAATTCTATTACTGAAC--CAACATACGA 149
D.affinis          --------CATACATA-TT--TAC--------AGTTCTATTACTGAACGTGAACATATGA 137
D.azteca           TAAACATATGTACATA-TT--TACCAATTAACAGTTCTATTACTGAA---GAACATATGA 161
D.bifasciata       --------CGCTGACATTTTTTACTAATTAAAGATTCTATTTTCTAAC--GAACACATGA 144
D.guanche          --------CGCTGAG--TTTTTCCTAATTAAATATTCTCTCACCGAAC--GAACATATGA 156
                          *   **  * *          **** *      **    **** * **

D.pseudoobscura    TAAAATTGGTGAAAATAATTGTCGGGAATCCCCATCC--GTTAAACACAACTTTTCCATC 207
D.persimils        TAAAATTGGTGAAAATAATTGTCGGGAATCCCCATCC--GTTAAACACAACTTTTCCATC 207
D.affinis          TAGAATTGCTGGAAATAATTGTCGGGAATTCCCATCCCTGTGAAACACAACTTTTCCATC 197
D.azteca           TAAAATGGCTGGAAATAATTTTCGGGAATCCCCATCCCTGTGAAACCCAACTTTTCCATC 221
D.bifasciata       T-CAAATATCAGAAATACCC-------ATCCTTGCC------ACA-GCAACTTTGCCATC 189
D.guanche          TGCAAATGCAGGAAATAATGGTCGGGAATCCTTATCC-TATAAAA-GCAACTTTTCCATC 214
                   *  **       *****          ** *    *      * *  ******* *****

D.pseudoobscura    ATTAAACATTATGATATTCAAGCCCC--------ACCTCAACAAACTTTCATCCACGAGT 259
D.persimils        ATTAAACATTATGATATTCAAGCCCC--------ACCTCAACAAACTTTCATCCACGAGT 259
D.affinis          ATTAAACATTGTGATATTCAAGCCCCGCCGACCCACCTCAACAAACTTTCATCCACGAGT 257
D.azteca           ATTAAACATTGTGATATTCAGGCCCCGCCGACCCACCTCAACAAACTTTCATCCACGAGT 281
D.bifasciata       ATTAAACATTGTGATATTCAAGTCCC--------ACCTCAACAAACTTTCATCCACGAGA 241
D.guanche          ATTAAACGTTGTGATATTCANGTCCC--------ACCTCAACAAACTTTTATCCACGAGA 266
                   ******* ** ********* * ***        **************** *********

D.pseudoobscura    ATCCCGAAA--CTTTTGGCAAAGTTTACGCATAATTTTTAACGATATTTTCCCGCATTTA 317
D.persimils        ATCCCGAAA--CTTTTGGCAAAGTTTACGCATAATTTTTGACGATATTTTCCCGCATTTA 317
D.affinis          ATCCTGGAAACTTTTTGGCAAAGTTTACGCATAATTTTTAACGATATTTTCCTGCATTTA 317
D.azteca           ATCCTGGAAACTTTTTGGCAAAGTTTACGCATAATTTTTAACGATATTTTCCTGCATTTA 341
D.bifasciata       GTCCTGAAAG-TTTTTGCCAAAGTTTACGCATAATTTTTAACGATATTTTCCCGCATTTA 300
D.guanche          GTCCTGAAA--CTTTTGGCAAAGTTTAAGCATAATTTTTAATGATATTTTCCCGCATTTA 324
                    *** * **   ***** ********* *********** * ********** *******

D.pseudoobscura    GCCTTCGAGTGGT-------------------GGTTGCTGGTGTTGTCTTCG---AGCACT 356
D.persimils        GCCTTCGAGTGGT-------------------GGTTGCTGGTGTTGTCTTCG---AGCACT 356
D.affinis          GCCTTCGAGTGCT-------------------GGTTGTTGATGTTGTCTTCGTGGAGCAGT 359
D.azteca           GCCTTCGAGTGCT-------------------GGTTGTTGATGTTGTCTTCGTGGAGCAGT 383
D.bifasciata       GCCTTCGAGTGTTTGTGGGTTGTTGGCGCTTGGTGGTTGGTGTTGTCTTCG---AGCACT 357
D.guanche          GCCTTCGAGAG---------------------GTGGTTGGTGTTGTCTTCG---AGCACT 360
                   ********* *                     ** * ** ***********   **** *

D.pseudoobscura    TTT----------------GTTGGCCTCC-ACGGATTCCAAATACGTTTCCGGCCAAACA 399
D.persimils        TTT----------------GTTGGCCTCC-ACGGATTCCAAATACGTTTCCGGCCAAACA 399
D.affinis          CCTCCTCC----ACGGTT--TTGCCCTCC-ATGGCCCCCAAATACGTTTCCGGCCAAACA 412
D.azteca           CCTCCTCCTCCAACCGTGAGTTGGCCTCC-ACGGATCTCAAATACGTTTCCGGCCAAACA 442
D.bifasciata       TTT----------------GTTGGCCCCCCACGGATCCCAAATACGTTTCCGGCCAAACA 401
D.guanche          TTC----------------GTTGGCCTCC-AGGGATCCCAAATACGTTTCCGGCCAAACA 403
                                      *** ** ** * **    **********************

D.pseudoobscura    ACTTTATTCAATTTCTCATATTGCATTGGCATTGG-TATTTTATTAAGCTGT-T-ATGCT 456
D.persimils        ACTTTATTCAATTTCTCATATTGCATTGGCATTGG-TATTTTATTAAGCTGT-T-ATGCT 456
D.affinis          ACTTTATTCAATTTCTCATCTTGGATTGGTATTGGTTACTTTATTAAGCTTT-TTTTGCT 471
D.azteca           ACTTTATTCAATTTCTCATCTTGCATTGGTATTGGGTATTTTATTAAGCTTT-TTATGCT 501
D.bifasciata       ACTTTATTCAATTACTCATATTG-----GCATTGG-TATTTTATTAAACTGTCTTATGCT 455
D.guanche          ACTTTATTCAATTTCTCATATTGCATTGGCATTTG-TATTTTATTAAGCTG--TTGTTCT 460
                   ************* ***** ***      * *** * ** ******** **    *  **

D.pseudoobscura    CTGAGCTCCT--GGATATTAAGATAAGACAACTTCCTGGTAAGTTTTCTTCCTGAATATT 514
D.persimils        CTGAGCTCCT--GGATATTAAGATAAGACAACTTCCTGGTAAGTTTTCTTCCTGAATATT 514
D.affinis          CTGAGCTCCT--GGATATTAAGATAAGACAACTTCAA----------CTTCGAGAATATT 519
D.azteca           CTGAGCGCCT--GGATATTAAGATAAGACAACTTCCC----------CTTTGTGAATATT 549
D.bifasciata       CTGAGCTACTCTGGATATTAAGATAAGACAACTTCCTGGCAAGTTTTCTTTGCGGTTATT 515
D.guanche          CTGAGCTCCTT--GATATTAAGATAAGACAACTTCTTGGTAAGTTTTCTTTCAGGATATT 518
                   ******  **   **********************            ***   *  ****

D.pseudoobscura    AAATAAATTTCGAGTGTTTAGTATTAAAATTTTATGGTAATCTCCGGCAAA-CGATTGAT 573
D.persimils        AAATAAATTTCGAGTGTTTGTTATTAAAATTTTATGGTAATCTCCGGCAAA-CGATTGAT 573
D.affinis          AAATAAATTTCGCGTGTTTGTTATTAAAATGTTATGGTAATCTCTGGCAAAACAATTGAT 579
D.azteca           AAATAAATTTCGCTTGTTTGTTATTAAAATGTTATGATAATCTTCGGCAAAACAATTGAT 609
D.bifasciata       AAATAAATTTCGCCTGTTTGTTATTAAAATTTTATGGTAATCTGCGGCAAA-CGATTAAG 574
D.guanche          AAATAAATTTCGCCAGTTTGTTATTAACATTTTATGGTAATCTCCGGCAAA-CGATTGAA 577
                   ************    ****  ****** ** ***** ******  ****** * *** *

D.pseudoobscura    --ATTTCTTTGCCAAACAAAAT------------TTGGATGGTTTT----TATTTTCGCA 615
D.persimils        --ATTTCTTTGCCAAACAAAAT------------TTTGATGGTTTT----TATTTTCGCA 615
D.affinis          --ATTTCTTTGCCAAGCAAAATATAGACATACAGATAGGAGGCTCCC-CGTATGTTTTCA 636
D.azteca           --ATT--------------------------GGACAGGCGG------CGTA--------- 626
D.bifasciata       ACATATCTTTGCCAAACAAGAT----------ACAAAAACAATCCGAAAGTATGCTTTGA 624
D.guanche          --ATTGCTTCGCCAAACAAGAT----------ATATCATAGA---GAAAGTGTGCTTTAA 622
                     **                                           *

D.pseudoobscura    AAAAATT------AAAATATTG-------CTCCCGT------------------------ 638
D.persimils        AAAGATT------AAAATATTG-------CTCCCGTTT---------------TCACAAA 647
D.affinis          AAACGGA------AAAATTTTAAACAAACCTCGCATAACAATACAAATCTTAATTGCAAA 690
D.azteca           ----------------------------CCTCG--------------------------- 631
D.bifasciata       AATTAATCTTTGAGCAATCTTTGA-----TTTCCGTAACGCAA--------------AAA 665
D.guanche          TATTTGT-----AACAATCTTTGG-----TTTCCGTAACGAAAT-------------AAA 659
                                                *

D.pseudoobscura    -------AAAAT---------AGA---TGACTTATTGAT------GCAGTAGCAATGGTA 673
D.persimils        AAAAAATAAAAT---------AGA---TGACTTATTGAT------GCAGTAACATT---- 685
D.affinis          AGAAAACAAAAT---------AGA---TGCCTTATCGCT------GCATTAACATT---- 728
D.azteca           ------------------------------------------------------------
D.bifasciata       GTTGCACAAAGTTTGCCAAGAAAAGTGTGTCAAACCGAAAG----GCGACAAAA------ 715
D.guanche          GTTGAACAAAGTT-----GGAAAA---TGTCCAACCGAAAGAAAGGCGACAAAATT---- 707

D.pseudoobscura    TAATTACAATTACATAATTACAAAGGCAATTTGCTTGTGCCTTTTTTAAAAATTATTGTA 733
D.persimils        TAATT-----TTCATAATTACAATTGCAATTT---------------------------- 712
D.affinis          TAATTGC-----CATAATTACCAGAGCAA------------------------------- 752
D.azteca           ------------------------------------------------------------
D.bifasciata       -AAAAA------TAAAGTTACGATAAAAAGTA---------------------------- 740
D.guanche          CGACAAG--CCGTAACGTAACGATAACAAGTC---------------------------- 737

D.pseudoobscura    TTTTCCTAGCCTAAAATTTTCCTCGTAAACAACATAGGGCTGCAAACGATTT----GGTT 789
D.persimils        -------------------TTCCTCGCAAACAACATAGGGCTCCAAACGAATT----GGTT 750
D.affinis          -----------------------------ATAACATAGGA----AAATA--TT----GCGC 774
D.azteca           ----------------------------------------------------T----GCGC 636
D.bifasciata       -------------------------GGGAGTAAAA-AACATTTAAAATACACACGAAATTC 775
D.guanche          -------------------------TGAAGGAACACGATAGCCAAAG-ACACTCGAAATTC 772

D.pseudoobscura    ATCGG-------AATGGA---AAAAAAA------TCACTTTGTGCGCGTAACAGCCCAAT 833
D.persimils        ATCGG-------AACGGA---AAAAAAAA-----TCACTTTGTGCGCGTAACAGCCCAAA 795
D.affinis          AT----------AACAGC---TCAAA-------------CTGTTCTCGTAACA------- 801
D.azteca           AT----------AACAGC---ACAAA-------------TTGTTCTCGTAACA------- 663
D.bifasciata       ATTCTGGCGAGCAACT-ATTGACAGAGTAATTCTTGTATTTTTACTCATAG-GAC----- 828
D.guanche          ATTC--------AATTGACTGACAAAATT-TTATTCTTTTTTTCTCACAGTGAA------ 818
                   **          **         * *             * * * * *   *

D.pseudoobscura    ------------CCAACA-----------AAAT----AGTTGAAAA-AAGTTGACCGTGG 865
D.persimils        TTGAGCTCGTTACCAACA-----------AAAT----AGTTGGAAA-AAATTGACCGTGG 839
D.affinis          --------------AACA-----------AAAT----ACTCGAAAA-AAGTTGTCCGAGA 831
D.azteca           --------------AACA-----------AAAT----ACTTGAAGT-AAGTTGTTCGAGG 693
D.bifasciata       --------------GGTACCT------GGGAAT-----CATGGGCA-AATCTGATGCAGC 862
D.guanche          --------------GATACATTTTCGTGTGAATTCAGACTCGTGCACGGCCTGATAGAGT 864
                                   *           ***          *        **     *
```

```
D.pseudoobscura  AAAGTGTGT----------------CAATGTCAAACCAAAAGGAAGACGACAAAAATTGA 909
D.persimils      AAAGTGTAT----------------CAATGTCAAACCAAAAGGAAGACGACAAAAATTGA 883
D.affinis        AAAGTGTGG----------------CAATGTCAAGCCAAAAGAAAGACGGCAAAAATTGA 875
D.azteca         AAAGTGTGT----------------CAATGTCAAACCAAAAGAAAGACG-CAAAAGTTAA 736
D.bifasciata     TAAGGGCCA--------------TACAAGTTC--CTCATTGGAGATGCTG--AGCTTAG- 903
D.guanche        TAAGGATCAACTGGTGGACCAACCGCAACTTCTGCGCATTAAAATCACTACAAACATGGA 924
                  ***                     ***  **    **         *    *   *

D.pseudoobscura  CAAGCCA---------------------TAAAGGTACGA-TAAAAAGTCAAGAGAAAATA 947
D.persimils      CAAGCCA---------------------TAAAGGTACGA-TAAAAAGTCAAGAGAAAATA 921
D.affinis        CGAGCCA---------------------GAAAGTTACGA-TAAAAATTCAAG--AAAGCA 911
D.azteca         --AATAA---------------------AAAAGTCAA------------AAG--AAAGCA 759
D.bifasciata     ----CCA-------------------CAGATGGTATCGACTTTGTTCCTGAG--AAAATA 938
D.guanche        AGAACCAAATAAAACTAAGAGCGAAGCAGATAGCCCCGAATATATTGTTGAG--AAAATA 982
                       *                      *  *                 **  ***  *

D.pseudoobscura  TT---CCAATGACTTTCGAACAAATTTATTCCAT---TGGCCAACACGATT-GTA----- 995
D.persimils      TT---CCAATGACTTTCGAACAAATTTATTCCAT---TGGCCAACACGATT-GTA----- 969
D.affinis        AT---TCAAGGGCCCTCGA---AATTCACAACAT---TGGCC--TTTGATTTGTACT--- 957
D.azteca         AT---TCAAAGCCTCTCGA---AATTCACAACAT---TGGCC--TTTGATTTGCATT--- 805
D.bifasciata     ATCGGTCAGCGAAAACATAAGGGCAATGTCGAATATCTGGTCA-AGTGGTT-GTACTACC 996
D.guanche        GTCGGTCAGAGAACTCAGAAGGGATGCGTCGAATATTTTGTCA-AATGGTT-GAATTACC 1040
                  *    **  *       *             **   * * *     * ** * *

D.pseudoobscura  --------GTCCATA-----AATATTCATGGTAAATTGAA---CTATTGAATGTTTTGTT 1039
D.persimils      --------GTCCATA-----AATATTCGTGGTAAATTGAA---CTATTGAATGTTTTGTT 1013
D.affinis        -CGCAGAGGACAGTA-----AATATTCGTGGTAAGTTGAA---CTATTGAATTCGTTGTT 1008
D.azteca         -TGCAGAGGTCAGTA-----AAAATTCATG-TAAGTTGAA---CTATTGAATGCGTTATT 855
D.bifasciata     CCGATGAGGATAATACGTGGGAACTTCCCAGCAGCCTGGATGGCTGT-GAACATCTGATT 1055
D.guanche        CAGAAGCGGATAATACTTGGGAACTTCCCAGCGACCTGAA---CTAT-GACCATCTGATC 1096
                         *    **      *  ***         ** *   ** * **     *  *

D.pseudoobscura  GTACCCCAACTGCTGCATCTGGAAATCATTAGAAGCATGGGAAAATCGGATGAAG----- 1094
D.persimils      GTACCCCAACTGCTGCATCTGGAAATCATTAGAAACATGGGAAAATCTGATGAAG----- 1068
D.affinis        TT-------------CATTTG-------TT------------------------------ 1018
D.azteca         TT-------------CATTTT-------TT------------------------------ 865
D.bifasciata     GCCGCCTAT------CATCTGCAACA-----CGA-------------------------- 1078
D.guanche        GCCGCTTAT------CATACTCAACAGAGTGTGAAGCGTCCATTCACTGTTGAGAAAATA 1150
                                ***

D.pseudoobscura  -----------------CGAAGGCCCATTCTAGTTCCTTATCGGAGATGAGACTT----- 1132
D.persimils      -----------------CGAAGGCCCATTCTAGTTCCTTATCGGAGATGAGGCTT----- 1106
D.affinis        -----------------CAAA------TTC--GTACCATACCCCAG---CGGCT------ 1044
D.azteca         -----------------CAAGG----TTC--G---------------------------- 874
D.bifasciata     -----------------CATTGATAA---------------------------------- 1087
D.guanche        ATCGGCCATAGAACTCTCATGGGTAATGTCGAATATCTTGTCAAGTGGTTGAATGTCTCC 1210
                                  *

D.pseudoobscura  ------------------------------------------------------------
D.persimils      ------------------------------------------------------------
D.affinis        ------------------------------------------------------------
D.azteca         ------------------------------------------------------------
D.bifasciata     ------------------------------------------------------------
D.guanche        GAAGAGGGTAGTACTTGGGAACAACCCAGTAGCCTGGTCTGTGACCATTTGATCGCCGCC 1270

D.pseudoobscura  ------------ATCTTCAACACAGC-ATTAAAGAAACCAACCAAAACGTACGATTGAAA 1179
D.persimils      ------------ATCCTCAACACAGC-ATTAAAGAAACCAACCAAAACGTACGATTGAAA 1153
D.affinis        -------------GCCTCTACAAATTAATTAGAAACATGGAC--AAATCTGCAAT----A 1085
D.azteca         -------------GCATCTACAAATTTATTAGAAACATAGAA--AAATCTGCAAT----A 915
D.bifasciata     -----------AGAACTGAACGAATT--GTACGAGTGCAAGGCAAAA----CGATTGAAA 1130
D.guanche        TATAATCTTCAAGACCTAAACGATGT--GTACGAAAAGAAAGCAAAA----CGATTGAAA 1324
                                *  **        **             ***    * **    *

D.pseudoobscura  GTTGATCCCGCTGTAGTAATTGATTATCCTTTCAGTCGTGGTTTCAAAGCTGAACAGATG 1239
D.persimils      GTTGATCACACTGTAGTCATTGATAATCCTTTCAGTCGTGGTTTCAAAGCTGAACAGATG 1213
D.affinis        GTCAAATCTGC-----------ATAACCCTTTCAGTCTTGGTTTCAAGGCTGAACAGATA 1134
D.azteca         GTCA---TTG------------ATAACCCTTTCAGTCGTGGTTTCAAGGCTGAACAGATA 960
D.bifasciata     GTCGATCCCACTGCAGTCATTGACAATCCCTTTAATCGTGGCTTCGAGGCTGAAAAGATA 1190
D.guanche        GTTGACCCCACGCCAGTCATTGATAATCCTTTTAGTCGTGGCTTTGAGGCTGAAAAAATA 1384
                 **                    *  * ** ** * ** *** **  * ****** * **

D.pseudoobscura  CTCGAAGGTTTCA-AAAAGGGAGAACAAATATCGCTTCTGATCAG--------GTCTATG 1290
D.persimils      CTCGAAGTTTTCA-AAAAGGGAGAACAAATATCGATTTTGATCAG--------GTCTATG 1264
D.affinis        CTCGAAGGTTTCA-AAAAGGGAGAAAAATCACAGTTTCTGAGCAATTTTTATCATCTGCA 1193
D.azteca         CTCAAAGGTGTCACAAAAGGGAGAAAAAGTACAGTTTCTGATCAA------------GCA 1008
D.bifasciata     CTCGAAGGTTTCA-AAAAGGGCGAACAAATATCGTTTCTGATCAAGTTTAGTGATCTGGT 1249
D.guanche        CTCAACAGTTTCA-AAAACGGAGAAGAAATATCGTTTCTAATAAAGTTTCGTAATCTGGA 1443
                 *** *   * *** **** ** *** **  *  * ** * *  *

D.pseudoobscura  TCTGTATCTAG-GCTTCG-----------CCTATGTGGAAATACCCCACACGGTCTTCAA 1338
D.persimils      TCTGTATCTAG-GCCACA-----------CCTATGTGGA---ACCCCACACGATCTTCAA 1309
D.affinis        GCTGCCGCAAGAGGATCAATTCGGCATAGCCCATGTGGAAATACCCCAAATGGTCTTTAA 1253
D.azteca         TCTGCCGCAAATGGTTCAAT-------------------------CCAAAACGTCTTTAA 1043
D.bifasciata     GCTGCCGCAAATGGTACCGTCGTACGTGGCCTATGTGGAAATACCTCANATGGTCTTTGA 1309
D.guanche        GCTGCCGCTAATGATCAAGTCCTACGTGGCCTATGTGGAAATACCCGACATGGTCTTTAA 1503
                  ***   * *  *                                  * *   ****  *

D.pseudoobscura  GTTCTACGAAAAGAATTGCCAATTTGCAAAACTTAATAAGGA----------------TT 1382
D.persimils      GTTCTACGAAAAGAATTGCCAATTTGCAAAACTTAATAAGGA----------------TT 1353
D.affinis        GTTCTATGAAAACCGTTAAAAGTTCATAAATCTACACGAGGA----------------TT 1297
D.azteca         GTTCTATGAAAACCGTTGCAAATTCATAAAACTATACCAAGA----------------TT 1087
D.bifasciata     ATTCTACGAAAAGAAATGCCACTTTATTAAACTCAATTAGCATTAAATGAAGAATGAATT 1369
D.guanche        GCACTACGAAAAGACATGCACATTTGTAACACCTAAATAGCA---------------ATC 1548
                    *** *****    *     **    *  *   *  *  *                *

D.pseudoobscura  GAATGAAAAACAAGACATTGTGTTGGACGTT-------------------TTGGGCACTT 1423
D.persimils      GAATAAAGAATA---------GTTA-----T-------------------TTAAA---TT 1377
D.affinis        GAATAAAGAA-------------------------------------------------- 1307
D.azteca         GAATGAAGAA-------------------------------------------------- 1097
D.bifasciata     GAATGAAGAAGGTAATCAAAAAGTATTTAAT-AATTTGTATACACAATTGTCTAATTGTT 1428
D.guanche        TA-TGAAGAAGGTAATATACAGGTTTTTTTTAATTTATCCAAACTAATGTCTAAAAATT 1607
                  * * ** **

D.pseudoobscura  TTTTCGTCAA----------AATTGCCT--------------TAAGATTCTTAGCAGATA 1459
D.persimils      TGTTCCTCAA----------AATTGCCT--------------TAAGATTCTGAACAGATA 1413
D.affinis        --------GT----------AG-------------------------------------- 1311
D.azteca         --------GA----------AGCAGCTG--------------CAAC-----------GTG 1114
D.bifasciata     TTGCAATTATTTAACAGCTCAATCGTTTGGTTACTACACAAATCAGCTGCTGTCAACGCA 1488
D.guanche        TCCTTATTATTTAACAGATCAATCGCTTGGCCACAACCCAAATAATCTGC-GGCAAAGCA 1666
                                     *

D.pseudoobscura  ---AATAACACCAAATCCTAATATGTATTATATTA--ATTTTTAT-TCTTATTCCATTAA 1513
D.persimils      ---AATAAGACCAAATCCGAATATGTATTATATTA--ATTTTTAT-TCTTATTCCATGAA 1467
D.affinis        ---------GCCAAAACCTAAT----CTTATATTC--ATATTGCTGTCGTCTTTCATAAA 1356
D.azteca         ---AATAAGGCCAAAACCCAAT----CTTATATTC--ATTTTGCTGTCGTCTTCCATGAA 1165
D.bifasciata     ---AATAAGGCCTACATCTAAT----ATTATATTATAATTTTAAG--CTTCTTCCATAAA 1539
D.guanche        CTGAACAATTCCTAAATCGAAT----ATTATTTTT--ATTTGAAG--TTCTTT-----AA 1713
                           ** *   * ***     **** **   ** *          **     **

D.pseudoobscura  ACTGTTGTACTGCAG-TTCCATTTGTTGTTTCTTTTATTTTACACATATTTGCACCATTA 1572
D.persimils      ACTGTTGTACTTCAG-TTCCATTTGTTGTTTCTTTTATTGTACACATATTTGCACCATTA 1526
D.affinis        G--GATCTACAACAT-CGCCATAT-TTG---------------GCATATTTGCATCATTA 1397
D.azteca         G--GATCTGCAACAT-CGCCAT---TTG------------------TTTTTGCACCATTA 1201
D.bifasciata     A--GATATTCAAGATATTCCATTTGTTTTTTGTCC---------TTTATTTGCACCATTA 1588
D.guanche        A--GATGTTCAACGT-TTGCACTTGATTTCTA-----------------TTGCA---TTA 1750
                    * * * *         **     *                      *****   ***

D.pseudoobscura  AATTGTGCCACTTTACGCAACATAATAAAAGGAAAA-A--TGCCAA--CTTTTAAGTGAT 1627
D.persimils      AATTGTGCCACTTTACGCAACATAATAAAAGGAAAA-A--TGCCAA--CTTTTAAGTGAT 1581
D.affinis        AATTGTGCCACTTTACGCAGGATAATAGATGGAAAA-AAATGCCAACATTTTTAAGTGAT 1456
D.azteca         AATTGTGCCACTTTACGCAGGATAATAAATGGAAAA-AAATGCCAA-ATTTTTAAGTGAT 1259
D.bifasciata     AATTGTGCCACTTTACGCAA---AATAGAAGGAAAAGAAGTGCCNA--CTTTTAAGTGAT 1643
D.guanche        AATTGTGCCACTTTGCGTAACATAATAGAAGGAAAT-AAATGGCAA--CTTTTAAGTGAT 1807
                 ************** ** *    **** * *****  *  ** * *   ***********

D.pseudoobscura  TTTCGAGGCGTGTTTGTTCCGGGTGCCCCC--GAGTAAACATTCCTAATTGCAACAACAA 1685
D.persimils      TTTCGGGGCGTGTTTGTTCCGGGTGCCCCC--GAGTAAACATTCCTAATTGCAACAACAA 1639
D.affinis        TTTCGGGGCGTGTTTGTTCCGGATGCCCCCC-GTGTAAACATGCCTAATTGCAACAACAA 1515
D.azteca         TTTCGGGGCGTGTTTGTTCCGGATGCCCCCCCGTGTAAACATGCCTAATTGCAACAACAA 1319
D.bifasciata     TTTCGGGCCGTGTTTGTTCCGAGTGCCCCC--GTGTAAACATTCCTAATTGCAACAACAA 1701
D.guanche        TTTCAGGGCGTGTTTGTTCCGGGTGCCCC---GTGTAAACATTCCTAATTGCAACAACAA 1864
                 ****  * *************  ******   * ******** *****************
```

Thursday, March 22, 12

```
D.pseudoobscura     CA-------TCGACTATG--GGGCAGG----CACCAAACAGAAG----------AGGCTC 1722
D.persimils         CA-------TCGACAACG--GGGCAGG----CACCAAACAGAAG----------AGGCTC 1676
D.affinis           CA-------TCCTCGACGCGGGGCAGG----CACCAAACAGAGG------CCAAAAGCTC 1558
D.azteca            CA-------TCCACAACGCGGGGCAGG----CACCAAACAGAGGAAGAGGCCAAAAGCTC 1368
D.bifasciata        CA-------TTCACAACG--GGGCAGGAGGGCACCGAACAG----------------C-C 1735
D.guanche           CGGGGAAGGCTCCAAACA--GGCGAAGAGG----CAAAGAG----------------CGC 1902
                    *              *    **  * *       * ** **                * *

D.pseudoobscura     GAGG-AATTTACAGCACGGGAAAATGAAGACAAAATATTTGCATTCCTTTTTTTT----- 1776
D.persimils         GAGG-AATTTACAGCACGGGAAAATGAAGACAAAATATTTGCATTC-TTTTTTTT----- 1729
D.affinis           GAGG-AATATACAACATGGGAAAATGAAGACAAAATATTTGCATTCCTTTTTTTTCTTGT 1617
D.azteca            GAGG-AATGTACAACATGGGAAAATGAAGACAAAATATTTGCATTCCTTTTTTTTCTTGC 1427
D.bifasciata        GAGGCGACGTACAGCACGGGAAAATGAAGACAAAATATTTGCATTCCTTTTTTTC----- 1790
D.guanche           GAAG-AATGTTCAGCACGGGAAAATGAAGACAAAATATTTGCATTCCTTTTTTCT----- 1956
                    ** *  *  * ** ** ***************************** ******

D.pseudoobscura     ---TTGTTTCTCTCTTTTGGCCCCCC--GTGGTGGATGGGGCGTGCCTGGTC----GGCC 1827
D.persimils         ---TTCTCTCTCTCTTTTGGCCCCCC--GTGGTGGATGGGGCGTGCCTGGTC----GGCC 1780
D.affinis           TTTTTGTTTCCCTCTTTTGGGCCCCC-AATGGTGGATGGGGCGTGGCTGGTCGGTCGGCC 1676
D.azteca            TTTTTGTTTCCCTCTTTTGGGCCCCCCAGTGGTGGATGGGGCGTGGCTGGTC----GGCC 1483
D.bifasciata        -------TCCC-TCTTTTGGCCCCCCCCCCAGTCGATGGGGCGTGGCTGGTC----GGCC 1838
D.guanche           -------CCCCATCCTTTGGCCCCC-----AGTGGATGGGGCGTGGCTGGTC----GGCC 2000
                             *  ** ***** ****      ** *********** ******    ****

D.pseudoobscura     AGTGGCCTATATTTGAGTCCGTTATTCAGCGTTTTGGTTT-GGTTTGTTGCGTTACTCCC 1886
D.persimils         AGTGGCCTATATTTGAGTCCGTTATTCAGCGTTTTGGTTT-GGTTTGTTGCGTTACTCCC 1839
D.affinis           AGTGGCCTATATTTGAGTCTGTTATTCAGCGTTTTGGTTTAGGTTTGTGGCGTTACTCCC 1736
D.azteca            AGTGGCCTATATTTGAGTCGGTTATTCAGCGTTTTGGTTTAGGTTTGTGGCGTTACTCCC 1543
D.bifasciata        AGTGGCCTATATTTGAGTCTGTTATTCAGCGTTTTGGTTT-----TGTGGCGTTACTCCC 1893
D.guanche           AGTGGCCTATATTTGAGGCTGTT-TTCAGCGTTTTGGTTT------GTGGCGTTACTCCC 2053
                    ***************** * *** ****************      ** ***********

D.pseudoobscura     AACAGGCACGTA-GTA-----------GCACCAGCAG--CCGCCATAGGGAATGCTTTGT 1932
D.persimils         AACAGGCACGTA-GTA-----------GCACCAGCAG--CCGCCATAGGGAATGCTTTGT 1885
D.affinis           AGCAGGCACGTA-GTA-----------GCACCAGCAGCGCCACCATAGAGAATGCTTTGT 1784
D.azteca            AGCAGGCACGTA-GTA-----------GCACCAGCAG--CCACCATAGAGAATGCTTTGT 1589
D.bifasciata        AACAGGCACGTACATACTACATACATAGCACCAGCAG--CCGCCATAGAGAATGCTTTGC 1951
D.guanche           AGCAGGCACGTA-GTACCC--------GCACCAGCAG--CCACCATAGAGAATGCTTTGT 2102
                    * **********  **           **********  ** ****** **********
```

(b.)

```
bifasciata      MGKSDAAKGHTSSSLEMLSLATDGIDFVPEKIIGQRKHKGNVEYLVKWLYYPDEDNTWEL 60
guanche         MEEPNKTKSEADS-----------PEYIVEKIVGQRTQKGCVEYFVKWLNYPEADNTWEL 49
                * :.: :*..:.*            ::: ***:***.:** ***:**** **: ******

bifasciata      PSSLDGCEHLIAAYHLQHDIDK-------------------------------------- 82
guanche         PSDLN-YDHLIAAYHTQQSVKRPFTVEKIIGHRTLMGNVEYLVKWLNVSEEGSTWEQPSS 108
                **.*:  :******* *:.:.:

bifasciata      -------------ELNELYECKAKRLKVDPTAVIDNPFNRGFEAEKILEGFKKGEQISFL 129
guanche         LVCDHLIAAYNLQDLNDVYEKKAKRLKVDPTPVIDNPFSRGFEAEKILNSFKNGEEISFL 168
                             :**::** **********.******.*********:.**:**:****

bifasciata      IKFSDLVLPQMVPSYVAYVEIPXMVFEFYEKKCHFIKLN 168
guanche         IKFRNLELPLMIKSYVAYVEIPDMVFKHYEKTCTFVTPK 207
                *** :* ** *: ********* ***:.***.* *:. :
```
