# Supplementary figures and images for: Phylogenomic Analysis Reveals Dynamic Evolutionary History of the Drosophila Heterochromatin Protein 1 (HP1) Gene Family
Source: PLoS Genet. 2012 Jun 21;8(6):e1002729. doi: 10.1371/journal.pgen.1002729 (PMC3380853; doi:10.1371/journal.pgen.1002729)

Figure S4.

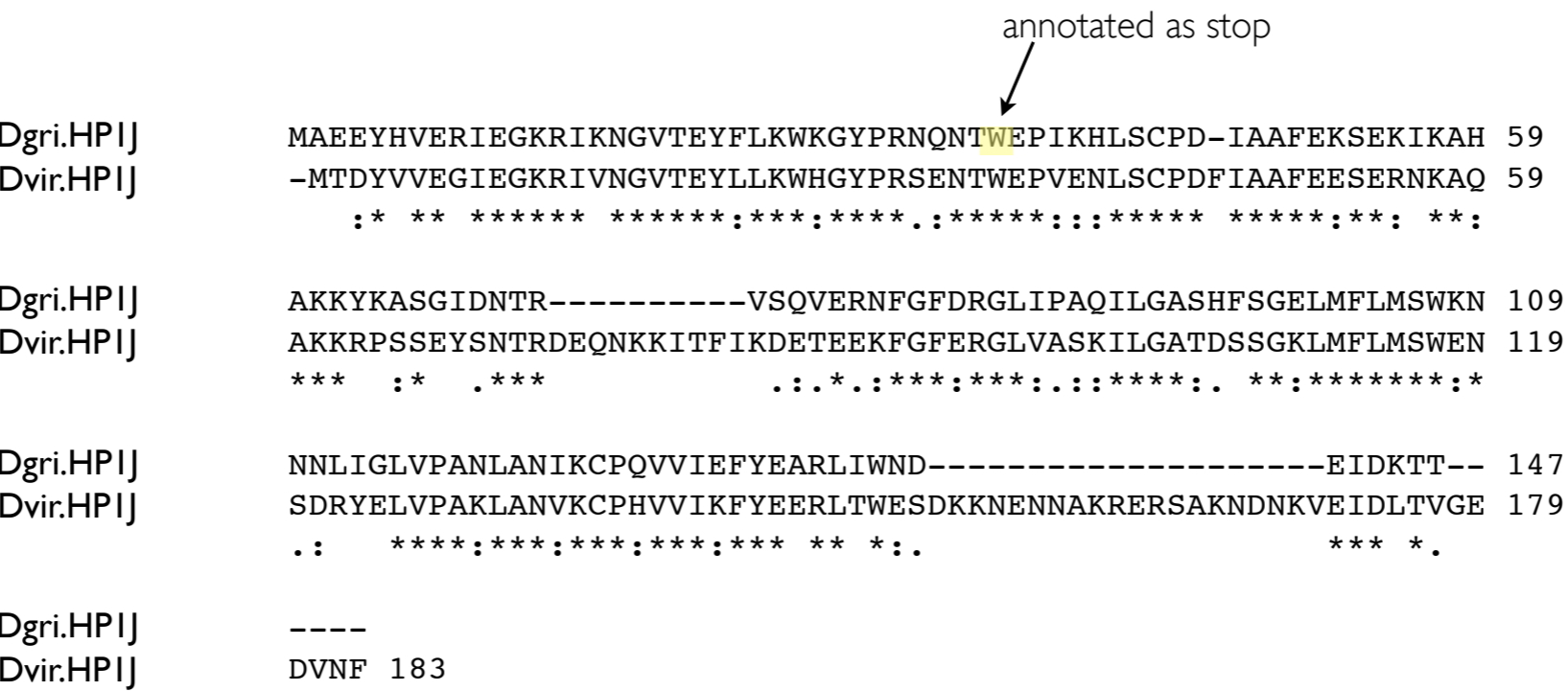

Supplement: Figure S4 — Protein alignment of HP1J from D. virilis and D. grimshawii. The residue annotated as a stop codon in the consensus genome sequence of D. grimshawii is highlighted in yellow. (PDF) [file pgen.1002729.s004.pdf]

Figure S5a.

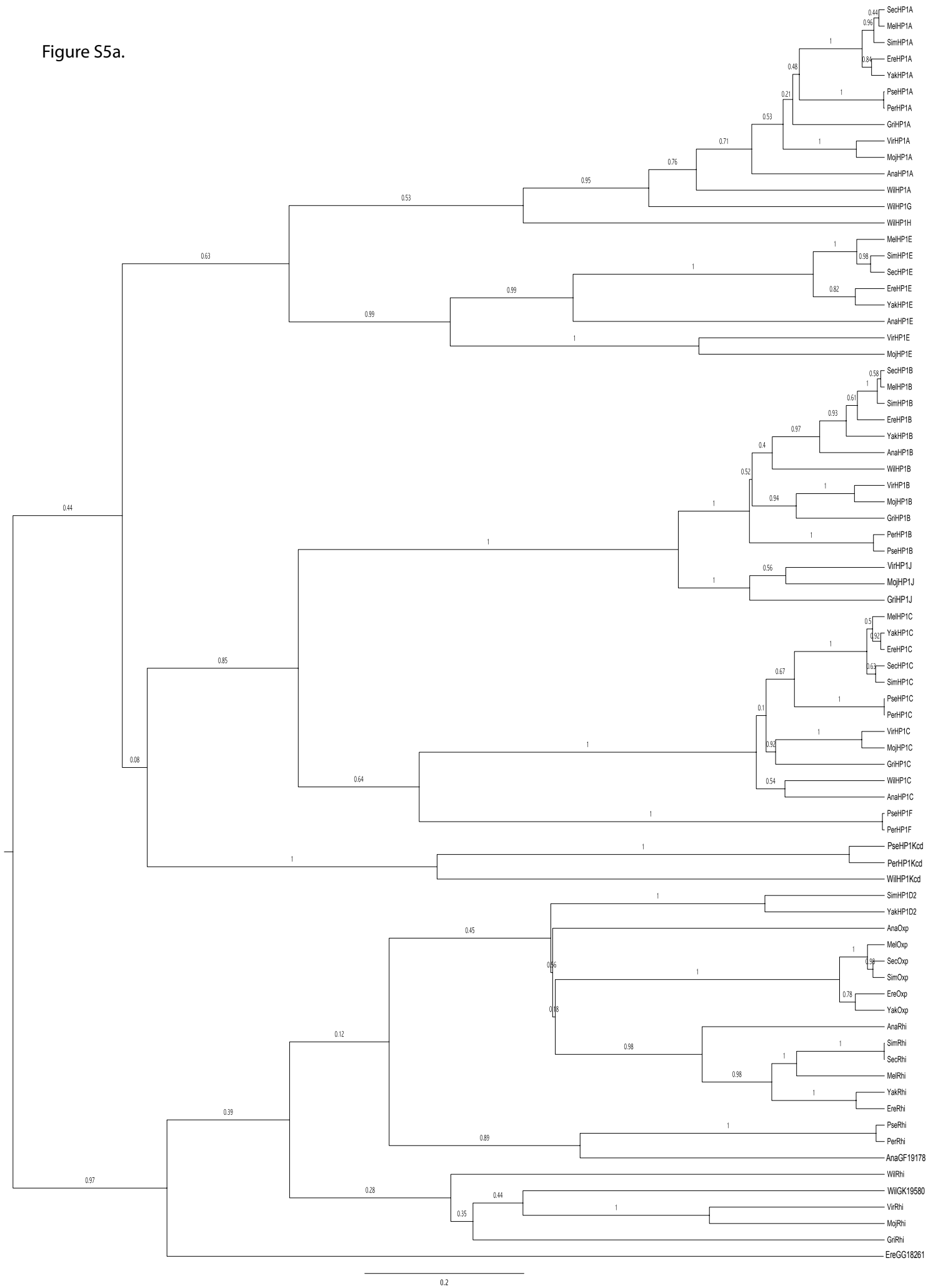

Figure S5b.

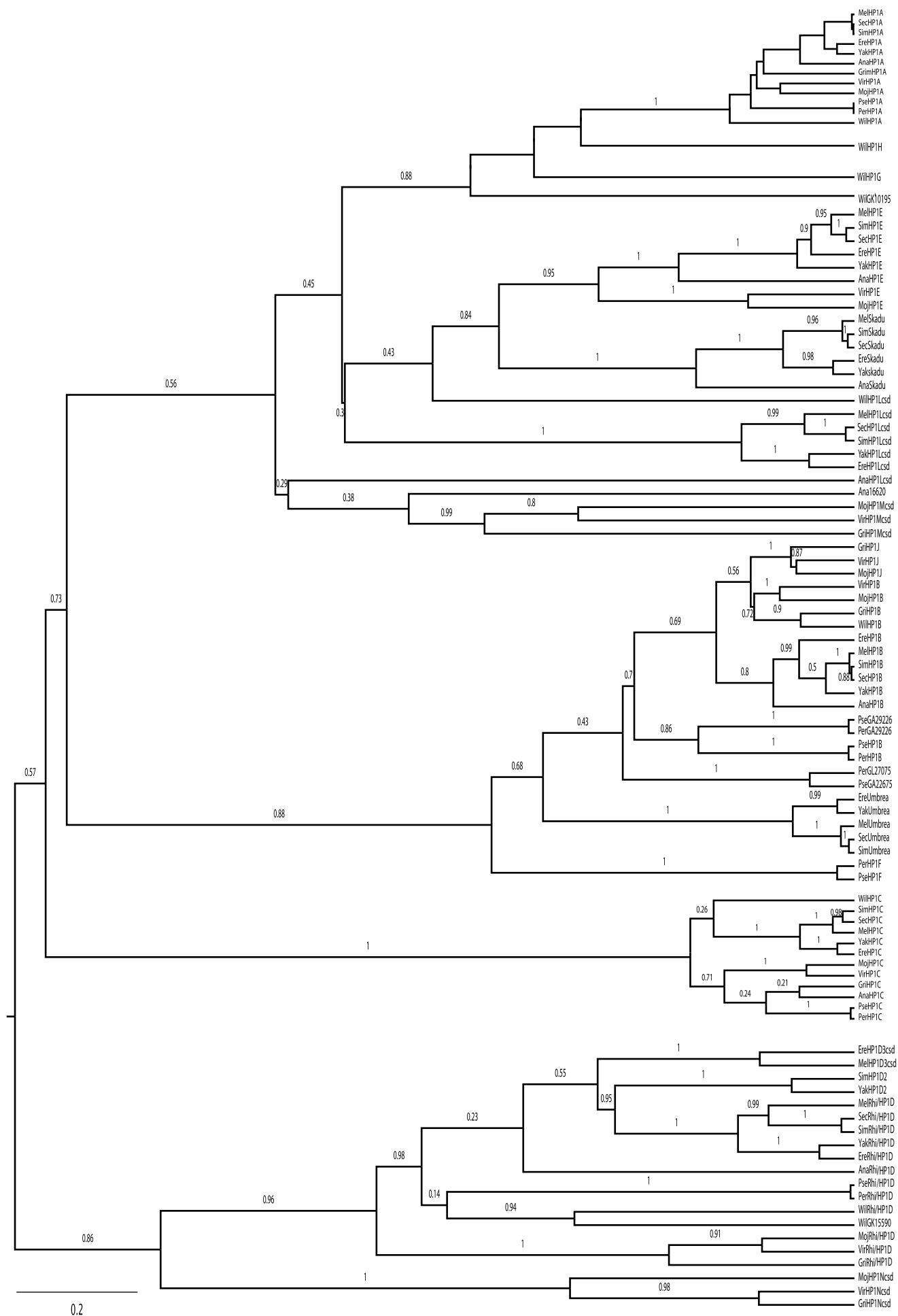

Supplement: Figure S5 — Phylogenetic trees with all support values reported (a) chromodomain (b) chromoshadow domain. (PDF) [file pgen.1002729.s005.pdf]

Figure S6a

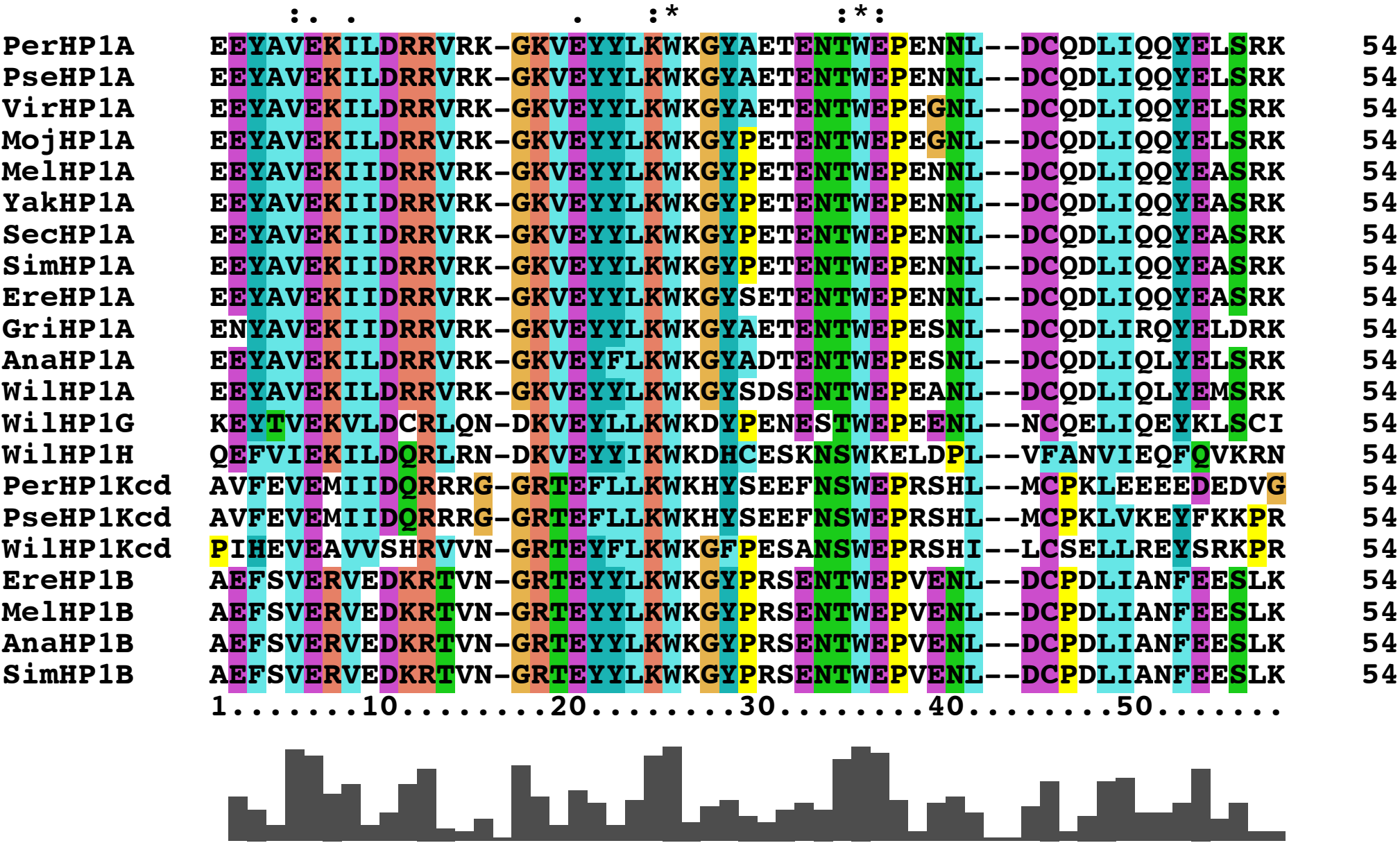

Figure S6b

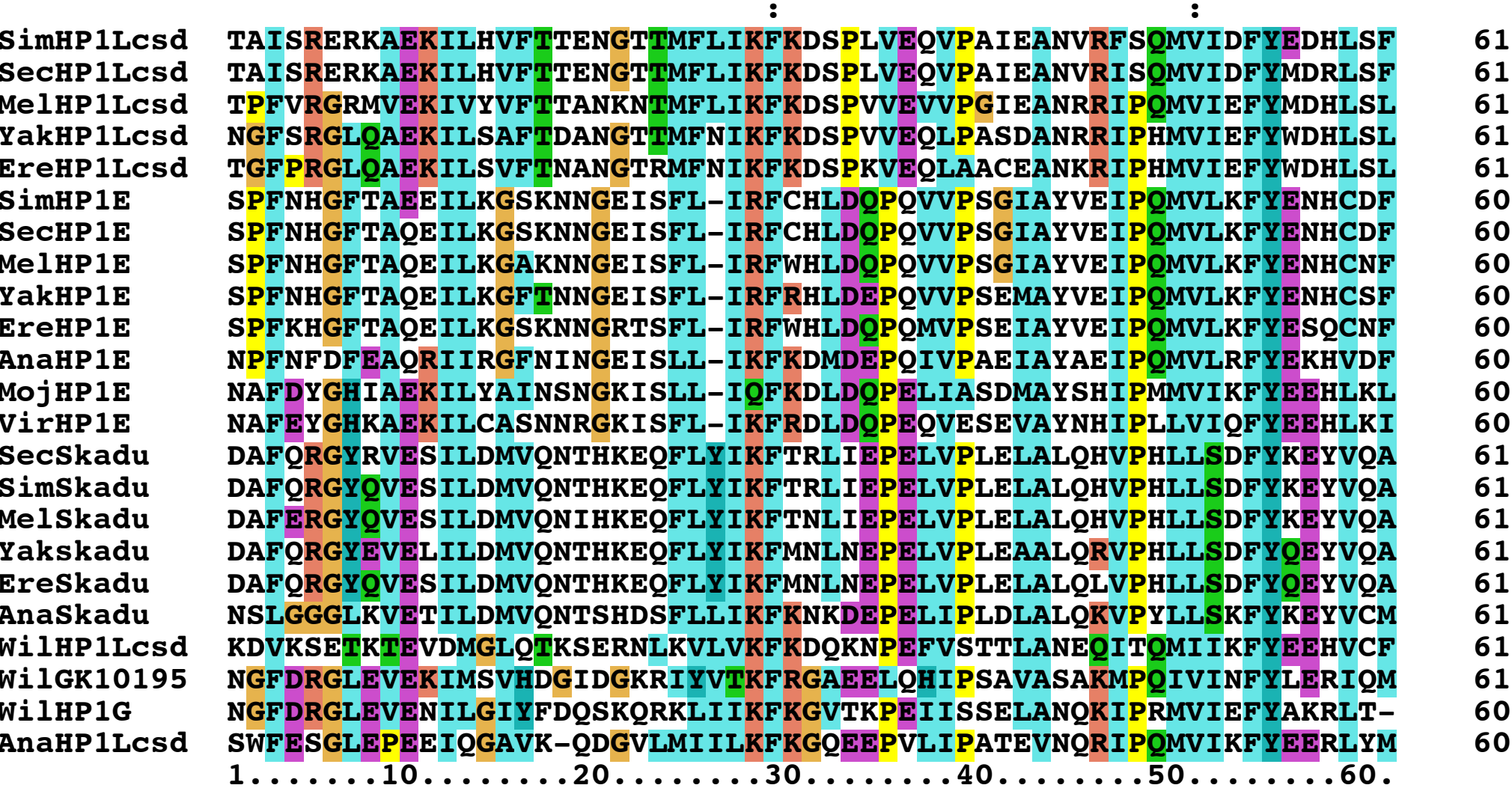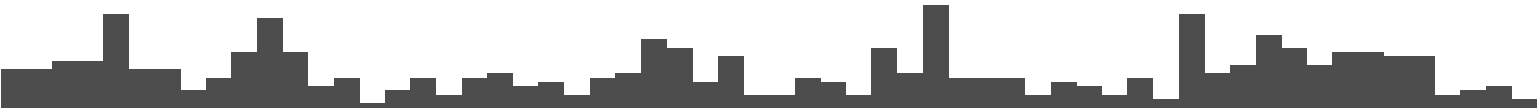

Supplement: Figure S6 — Amino acid alignments for the (a) chromodomain (b) chromoshadow domain. (PDF) [file pgen.1002729.s006.pdf]
